# Highlights lecture EANM 2015: the search for nuclear medicine’s superheroes

**DOI:** 10.1007/s00259-016-3423-4

**Published:** 2016-05-27

**Authors:** Andreas Buck, Clemens Decristoforo

**Affiliations:** 1 Department of Nuclear Medicine, University Hospital Würzburg, Würzburg, Germany; 2 Department of Nuclear Medicine, Medical University Innsbruck, Anichstrasse 35, A-6020 Innsbruck, Austria

**Keywords:** Highlights Lecture, 2015, EANM, Hamburg, Physics and instrumentation, Radiopharmacy, Molecular Imaging, Oncology, Radionuclide Therapy, Cardiology, Neurosciences

## Abstract

The EANM 2015 Annual Congress, held from October 10th to 14th in Hamburg, Germany, was outstanding in many respects. With 5550 participants, this was by far the largest European congress concerning nuclear medicine. More than 1750 scientific presentations were submitted, with more than 250 abstracts from young scientists, indicating that the future success of our discipline is fuelled by a high number of young individuals becoming involved in a multitude of scientific activities. Significant improvements have been made in molecular imaging of cancer, particularly in prostate cancer. PSMA-directed PET/CT appears to become a new gold standard for staging and restaging purposes. Novel tumour specific compounds have shown their potential for target identification also in other solid neoplasms and further our understanding of tumour biology and heterogeneity. In addition, a variety of nuclear imaging techniques guiding surgical interventions have been introduced. A particular focus of the congress was put on targeted, radionuclide based therapies. Novel theranostic concepts addressing also tumour entities with high incidence rates such as prostate cancer, melanoma, and lymphoma, have shown effective anti-tumour activity. Strategies have been presented to improve further already established therapeutic regimens such as somatostatin receptor based radio receptor therapy for treating advanced neuroendocrine tumours. Significant contributions were presented also in the neurosciences track. An increasing number of target structures of high interest in neurology and psychiatry are now available for PET and SPECT imaging, facilitating specific imaging of different subtypes of dementia and movement disorders as well as neuroinflammation. Major contributions in the cardiovascular track focused on further optimization of cardiac perfusion imaging by reducing radiation exposure, reducing scanning time, and improving motion correction. Besides coronary artery disease, many contributions focused on cardiac inflammation, cardiac sarcoidosis, and specific imaging of large vessel vasculitis. The physics and instrumentation track included many highlights such as novel, high resolution scanners. The most noteworthy news and developments of this meeting were summarized in the highlights lecture. Only 55 scientific contributions were mentioned, and hence they represent only a brief summary, which is outlined in this article. For a more detailed view, all presentations can be accessed by the online version of the European Journal of Nuclear Medicine and Molecular Imaging (Volume 42, Supplement 1).

## Introduction

From October 10–14, 2015, 5550 participants met at the 28th Annual Meeting of the European Association of Nuclear Medicine (EANM) in Hamburg, Germany. This is by far the largest European congress related to nuclear medicine and was chaired by Prof. Wim Oyen, representing EANM’s scientific committee, organizing a highly multidisciplinary programme comprising 425 oral and 1208 poster presentations. Besides regular participants, 775 professionals followed the congress programme online via the EANM live stream. More than 1000 attendees were coming from non-European countries, with strongest representations from USA, Japan, Australia, and Canada.

**Fig. 1:**
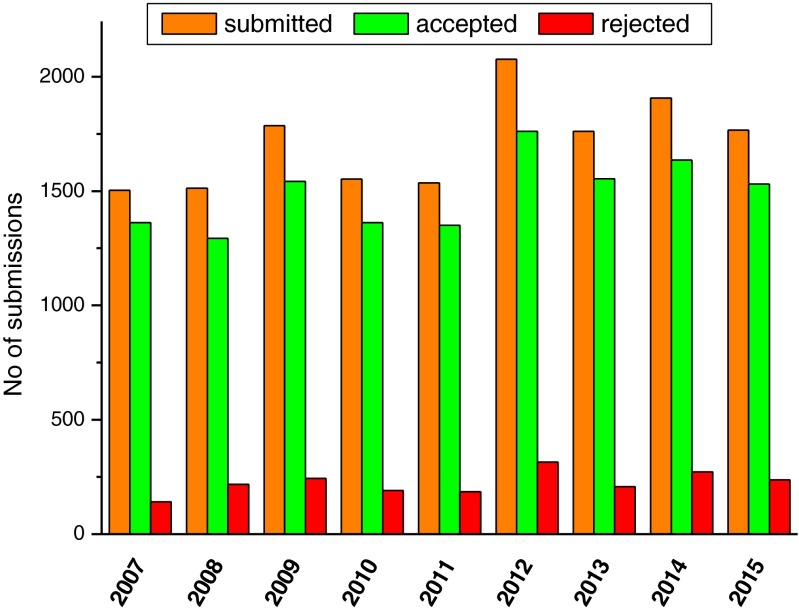
EANM annual meeting statistics: Submitted, accepted, and rejected abstracts for the scientific programme in recent years

Twenty sessions on continuing education (medical doctors and technologists), four plenary sessions, 24 symposia and pre-symposia surrounded the scientific programme. In total, 1767 abstracts for the scientific programme were received, 1530 abstracts were accepted (rejection rate, 13.4 %), the third highest number in the history of the meeting, only topped by last year and the 25^th^ EANM anniversary meeting in Milan 2012 (Fig. 1). In addition, 137 technologist’s abstracts were included in the programme.

**Fig. 2:**
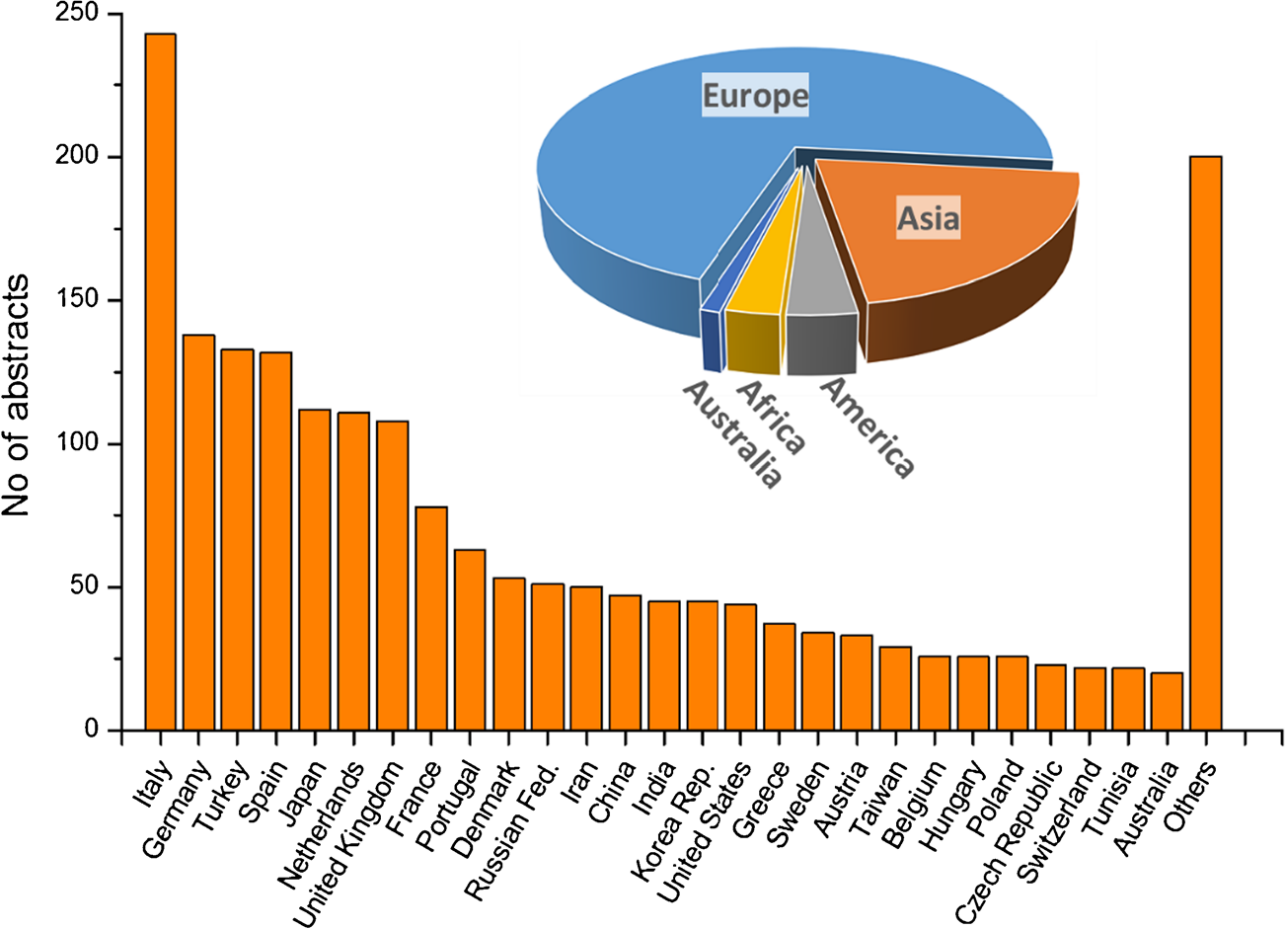
EANM’15: Abstracts per country and continent

In total, abstracts were submitted from 83 different countries worldwide (Fig. 2). The highest number of abstracts was received, as in the years before, from Italy (13.8 %), followed by Germany (7.8 %), Turkey (7.5 %), Spain (7.5 %), and then, as the first non-European country, Japan (6.3 %). By continent, 71.7 % of abstracts were from Europe, 20.7 % from Asia, 3.6 % from the Americas, 2.8 % from Africa, and 1.1 % from Australia.

**Fig. 3:**
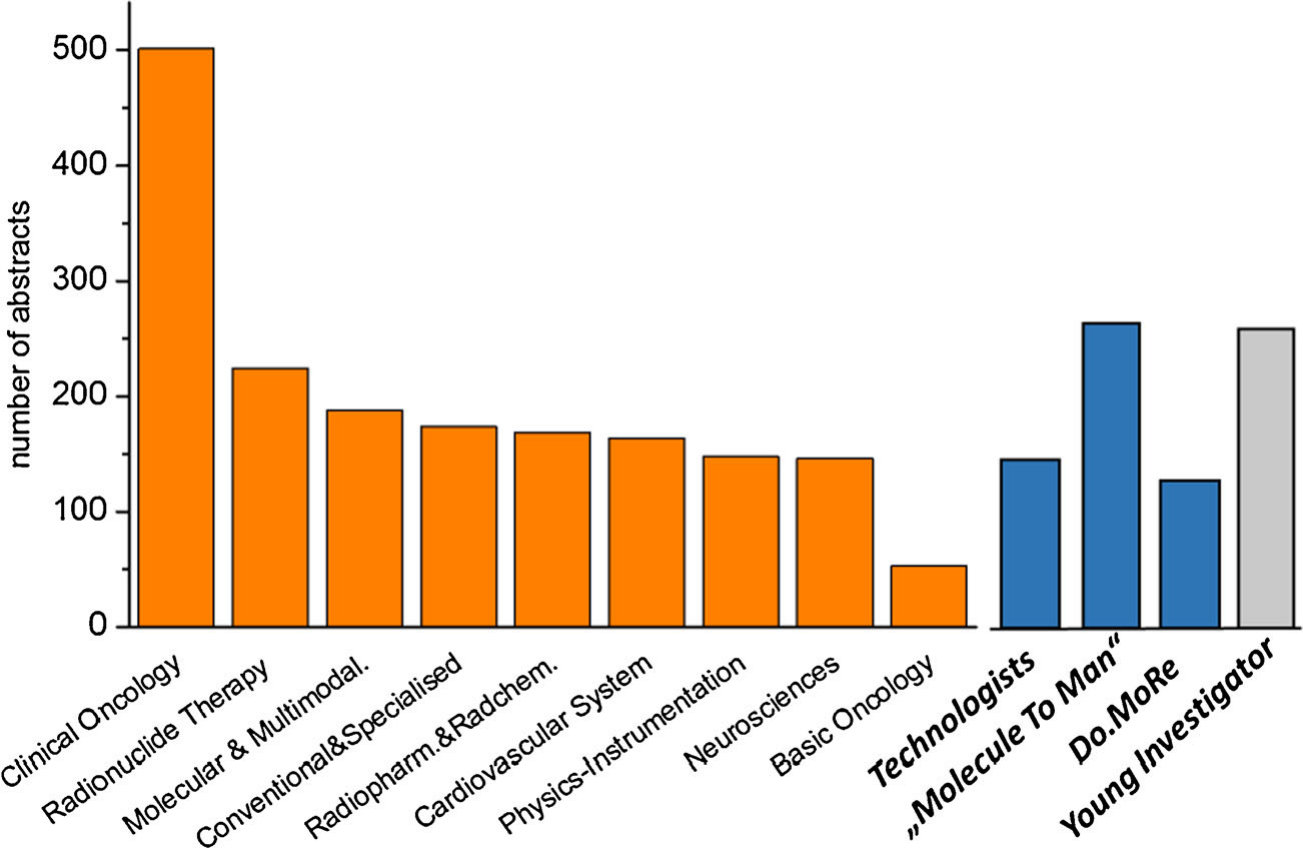
EANM’15: Abstracts per category

Multidisciplinarity has always been a core value of nuclear medicine and is reflected by looking at the scientific topics covered at the meeting. The abstracts were distributed in a wide range of topics including clinical and basic oncology, neurosciences, cardiovascular system, radionuclide therapy, conventional and specialized nuclear medicine, molecular and multimodality imaging, radiopharmacy and radiochemistry, and physics and instrumentation. The numbers of submitted abstracts per category are shown in Fig. 3. Oncological applications dominate nuclear medicine practice and research. A great majority of abstracts dealt with oncological science, led by clinical oncology with 501 abstracts, but also involves basic oncology (53 abstracts) and radionuclide therapy with more than 200 abstracts, not to forget that many oncological topics were covered in other categories such as radiopharmacy or physics. Other categories were comparably represented with about 150–190 abstracts in six categories showing the high research activity in a wide range of topics. Technologists contributed with their own programme including 137 abstracts. Special tracks stressed the translational aspect from molecule to man, which was introduced at last year’s meeting and was well accepted with 264 abstract submissions in this category. Multidisciplinary aspects of dosimetry and targeted radionuclide therapy in the DoMoRE track were also of focused on with more than 100 abstracts. Most promising for the future of nuclear medicine was the fact that more than 250 abstracts (15 %) were submitted by young investigators, indicating the interest and activity of young colleagues entering the field and becoming involved in scientific activities.

All abstracts were rated by expert reviewers and, independent of the topic, those with the best individual scores were considered for the Highlights Lecture. Having the honour and task to review these abstracts we realized the great potential of scientists and the great efforts they undertake to bring our discipline forward. This is really a heroic work, and representative for all “superhero” scientists we took the task to hunt for the best science as PETman (Andreas Buck) representing the clinical science, supported by his friend Robin (Clemens Decristoforo) looking into basic sciences and highlighting the translational aspects being representative for this year’s meeting.

From more than 170 excellent presentations received, which is highly acknowledged, only 55 were finally selected and included in the highlights presentation, often being only examples for a great number of similar and scientifically equally good submissions related to the limited time available. So follow PETman and Robin’s hunt for the best science at EANM’15 meeting in Hamburg.

## Oncology: diagnosis

### Prostate cancer

**Fig. 4:**
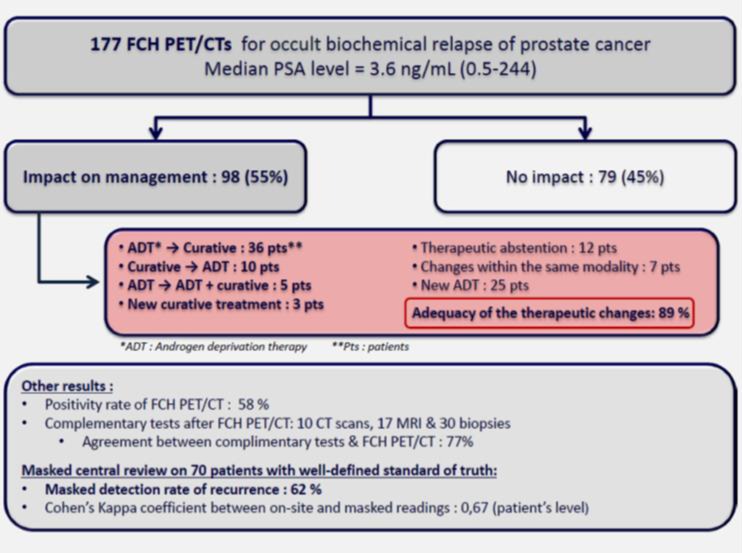
Gillenbert et al. [1] showed in a large prospective multicenter clinical trial comprising 177 patients with biochemical relapse from prostate cancer that ^18^F-Choline-PET/CT has a major impact on the therapeutic decision making process in 55 % of patients. An expert panel rated almost 90 % of therapeutic changes as adequate. However, a positivity rate of 58 % indicates that a significant proportion of patients has negative imaging results, and thus do not benefit from PET/CT imaging

Diagnostic imaging in oncology was fueled by a particularly large number of contributions addressing staging and restaging of prostate cancer. In a prospective multicenter trial, Gillenbert and coworkers reported on a series of almost 180 patients undergoing ^18^F-Choline-PET/CT for detecting biochemical relapse from prostate cancer [1]. This paper nicely showed the clinical relevance of this established imaging modality with a significant impact on therapeutic management in up to 55 % of patients (Fig. 4). However, the positivity rate of ^18^F-Choline-PET/CT was as low as 58 %, indicating that this modality is unable to detect the area of recurrence in more than 40 %.

**Fig. 5:**
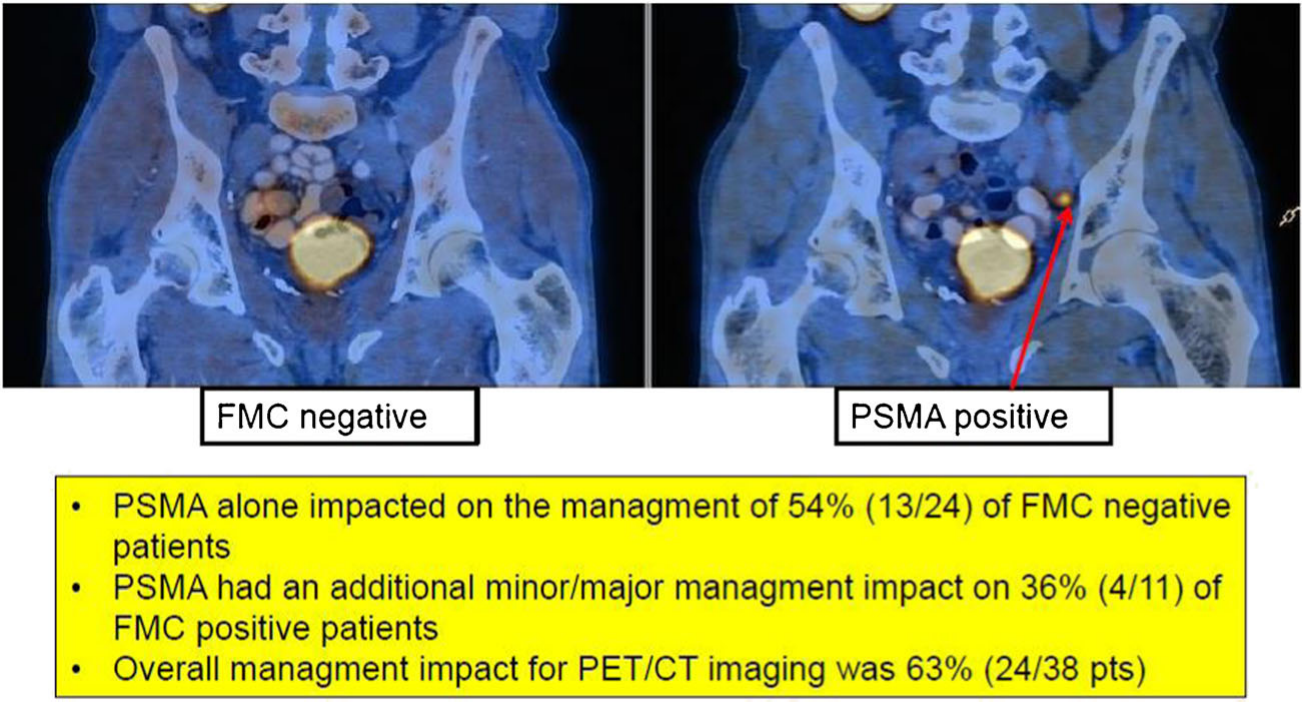
A variety of scientific presentations have shown superiority of PSMA-directed radiotracers as compared to choline-based PET tracers. Morighi et al. [2] have shown that PET sensitivity for detecting the anatomic site of biochemical relapse has doubled when ^68^Ga-PSMA is used as the PET radiotracer. The figure shows focally increased ^68^Ga-PSMA uptake in an iliac lymph node metastasis, whereas the corresponding ^18^F-Fluoromethylcholine-PET/CT scan showed no correlate of recurrent prostate cancer

This obstacle was specifically addressed by an Italian study (Morighi et al.) [2]. In a prospective trial comparing ^18^F-Fluormethyl-Choline (FMC) and ^68^Ga-PSMA-PET/CT, sensitivity has doubled using the PSMA-targeted approach as compared to FMC (Fig. 5). In this series, detection rate of FMC-PET/CT was as low as 32 and 66 % in the PSMA-PET/CT group. Two further clinical trials provided evidence that detection rates of PET imaging in recurrent prostate cancer can be further improved using additional targets. Zanoni et al. from the Bologna group investigated the amino acid based tracer ^18^F-FACBC [3]. In a comparative analysis, an improvement of sensitivity, as well as specificity versus ^11^C-choline-PET/CT has been shown. With a sensitivity of 37 % and a specificity of 67 %, the performance of ^18^F-FACBC seems to be in an intermediate position between choline-based and PSMA-targeted radiopharmaceuticals. An overall accuracy of 38 %, however, indicates an inferiority of ^18^F-FACBC compared to ^68^Ga-PSMA.

Targeting the bombesin receptor is another approach for specific imaging of prostate cancer. The concept of using receptor antagonists has shown success in overcoming limitations, especially in terms of side effects, but also resulting in improved targeting properties. In a presentation from Bakker et al. [4], first results in therapy-naïve prostate cancer patients with [^68^Ga]Sarabesin 3, a novel GRP receptor antagonist were reported. In this preliminary study three patients with prostate carcinoma confined to the prostate were included and imaged over a period of 3.5 h. No side effects were observed; pharmacokinetics were favourable with rapid predominant renal excretion and low background, indicating high metabolic stability. All patients showed focally increased uptake in the prostate, which correlated to tumour-GRP expression in autoradiography studies after prostatectomy, indicating the suitability of the tracer to image therapy-naïve primary prostate cancer, confirming the potential of GRP as an alternative to PSMA-targeting.

Assessing the androgen receptor status is another important field of PET imaging of prostate cancer, in particular for therapy response evaluation. A novel PET tracer, [^18^F]enzalutamide, was reported by Antunes et al. [5]. Also, in this case advantages could be expected using an antagonist to target the androgen receptor. In preclinical studies they showed that [^18^F]enzalutamide resulted in higher tumour uptake, higher tumour/plasma and muscle ratio combined with a high in vivo stability and lower urinary excretion as compared to [^18^F]FDHT, making this compound an interesting and highly promising candidate for translation into human studies.

Despite the success of [^68^Ga]HBED-CC-PSMA in the diagnosis of prostate cancer, there is still room for further development for wider availability of the methodology, enabling in particular centralized production of PSMA targeting PET ligands. In this respect, in this meeting three presentations are representative for new radiopharmaceutical developments towards PSMA targeting tracers labelled with longer lived radionuclides. Gourni et al. [6] presented a versatile NODAGA-PSMA conjugate suitable for labelling with ^111^In for SPECT, as well as ^68^Ga and ^64^Cu for PET imaging. NODAGA-Phe-PheD-Lys(suberoyl)-Lys-urea-Glu (VG66) showed high affinity and rapid internalisation on PSMA expressing LNCaP cells, when radiolabelled with all three radionuclides. Tumour uptake of ^68^Ga-VG66 (14.5 ± 2.9 % IA/g) in LNCaP xenografts at 1 h p.i. was comparable to ^68^Ga-HBED-CC-PSMA (15.8 ± 1.4 % IA/g) (*P* = 0.67), for ^111^In-VG66 28.5 ± 2.6 % IA/g were found. PET-images with ^64^Cu-VG66 at later time points showed wash-out from the kidneys, while tumour uptake still remained high. This holds promise to widen the applicability of the methodology towards SPECT and PET enabling centralized production and distribution using ^64^Cu-labelling.

**Fig. 6:**
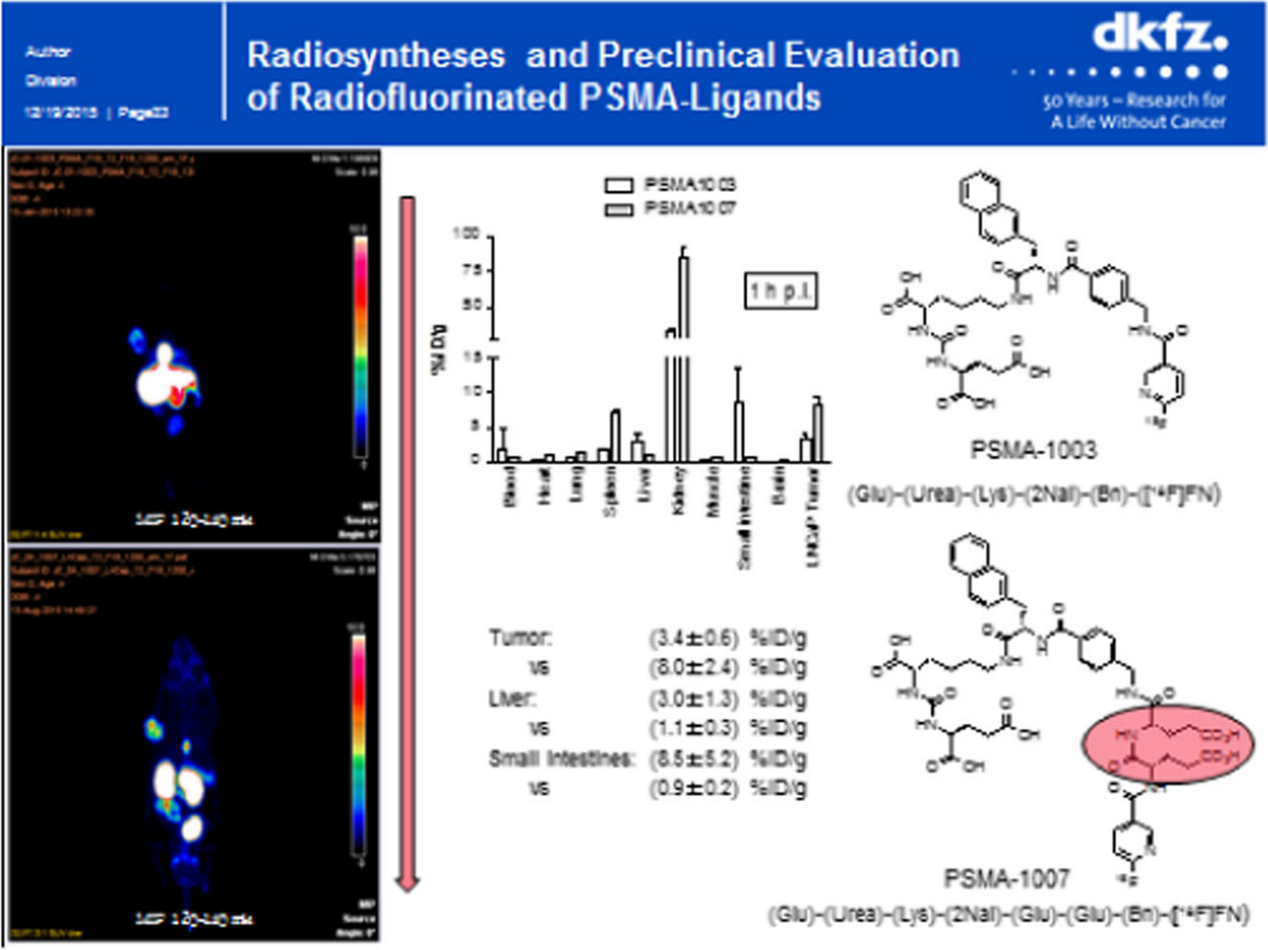
Development of novel ^18^F-PSMA ligands by Cardinale et al. [7]. Left: MIPs gained by PET imaging after injection (2 h p.i.) of PSMA-tracers shown on the right side in LNCaP tumour bearing mice. The arrow indicates the aim to improve the organ distribution of PSMA-1003 (upper part of the slide), resulting in PSMA-1007 (lower part of the slide). The red oval highlights the difference in both tracers. The diagram in the middle shows a comparison of the organ distribution of both tracers at 1 h p.i.

Two presentations dealt with [^18^F]-labelled PSMA-ligands. Cardinale et al. [7] reported on the radiosynthesis and preclinical evaluation of radiofluorinated PSMA-ligands (Fig. 6). They synthesized a series of novel fluorinated tracers, and optimized biodistribution by introducing a spacer directing pharmacokinetics towards renal excretion, resulting in PSMA 1007 with excellent imaging characteristics in LNCaP tumour bearing mice. Another approach was attempted by Boschi et al. [8]. They developed an a “one-pot” method for radiofluorination of the HBED-CC-PSMA precursor, currently used for ^68^Ga labelling, using aluminium [^18^F]fluoride (AlF), achieving high yields at high specific activities. In the LNCaP mouse model excellent image contrast was achieved.

The coming years will reveal which compound can be successfully translated into the clinical setting complementing the excellent results so far achieved with ^68^Ga-labelled PSMA radiopharmaceuticals.

### Neuroendocrine cancers

**Fig. 7:**
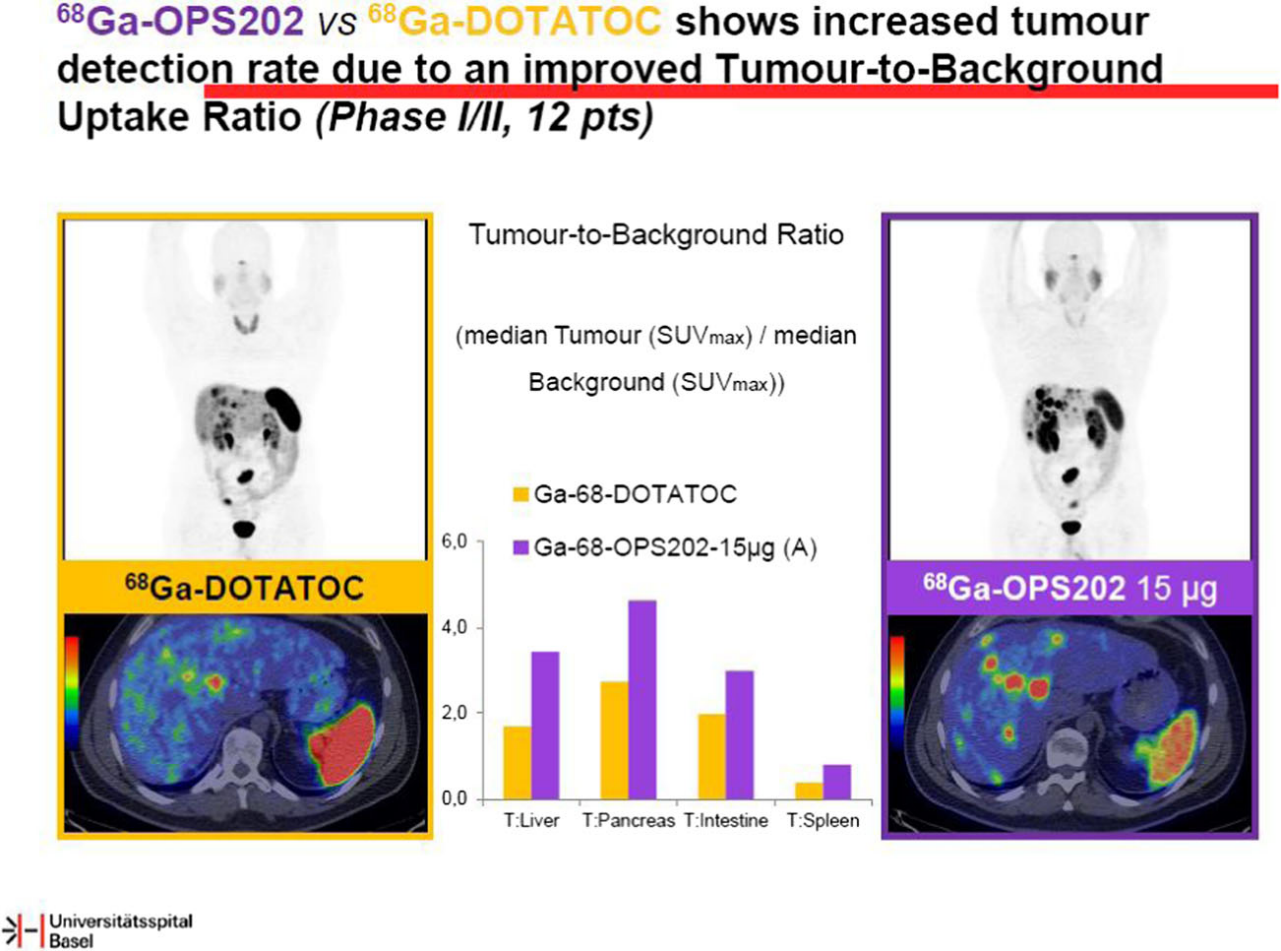
Use of somatostatin receptor antagonists instead of agonists results in an improvement of tumour-to-background ratio in neuroendocrine tumours. Nicolas and coworkers presented novel data on the utility of the SSTR antagonist ^68^Ga-OPS202 which showed an improvement in sensitivity (right) as compared to the standard imaging approach using radiolabelled SSTR agonists (^68^Ga-DOTATOC, left) [10]. Ultimately, in a theranostic setting, SSTR antagonsits may also improve therapeutic outcome after peptide radioreceptor therapy (PRRT)

Targeting somatostatin receptor expression for imaging neuroendocrine tumours represents a mainstay diagnostic test in daily clinical practice. Skoura and coworkers have looked at the impact of ^68^Ga-DOTATATE-PET/CT for managing patients with neuroendocrine tumours [9]. In a retrospective cohort comprising 1.258 PET/CT-scans, a major impact on the therapeutic decision making process has been described in more than 40 % (515 patients). This study nicely illustrates the clinical relevance of SSTR-directed PET imaging in patients with NET and suspicion on disease recurrence or progression. An interesting study by Nicolas et al. from the Basel group have presented novel data on radiolabeled somatostatin antagonists for PET imaging and radionuclide therapy [10]. Initial data from preclinical models, as well as clinical data indicate that ^68^Ga-OPS202 provides PET images with high tumour-to-background ratios and increased tracer retention as compared to ^68^Ga-DOTATOC, facilitating more sensitive tumour detection (Fig. 7). In addition, dosimetric analyses in preclinical animal models indicate an improvement of tumour-to-kidney dose ratio of more than 34 % and significantly enhance retention in tumour xenotransplants. Recently, the SSTR-specific theranostic approach has emerged as a superior imaging and treatment modality for NET patients. This novel approach using SSTR antagonists instead of agonists may further enhance the diagnostic accuracy of SSTR-directed molecular imaging and may result in improved therapeutic outcomes of patients with NETs treated with peptide radioreceptor therapy.

### Intraoperative imaging and navigation

**Fig. 8:**
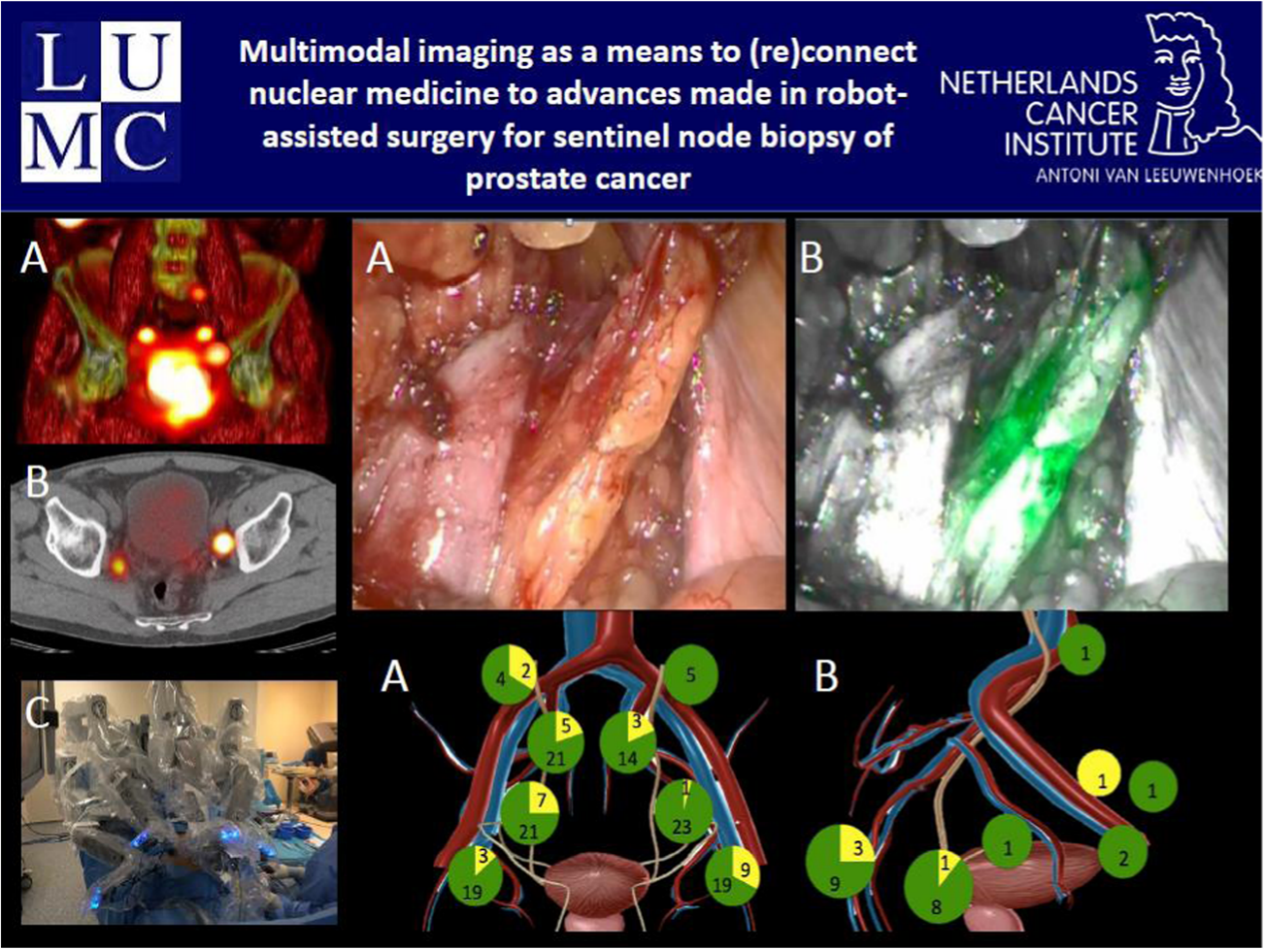
KleinJahn and coworkers [12] reported on an innovative approach for intraoperative imaging of sentinel lymph nodes (SLN) in patients with prostate cancer. They have shown the feasibility of SLN dissection using multimodal imaging. 1A, 3D volume rendering showing the SNs; 1B, fused SPECT/CT with SN; 1C, da Vinci Si robot with integrated fluorescence laparoscope; 2A, White light image illustrating the area harbouring the SN; 2B, fluorescence guidance clearly shows the contours of the SN; 3A, distribution of the sentinel nodes inside the ePLND area; 3B, distribution of the SN outside the ePLND area. In green the in vivo fluorescent SN and yellow the SN that were only radioactive in vivo

An increasing number of abstracts dealing with sophisticated imaging modalities for guiding surgical interventions were recognized during the meeting. Recent evidence that radioguided surgery can improve success rates, especially regarding detection of occult tumour lesions was described by Pouw et al. from Amsterdam [11]. Using a 3D-freehand SPECT device, successful detection of occult breast cancer lesions labelled with 125-Iodine seeds prior to the incision has been demonstrated. Whereas the vast majority of abstracts have focussed on lesion localization in breast cancer and melanoma, there were also contributions of radioguided surgery as integral part of more complex surgical interventions. Of note, Kleinjahn et al. presented an interesting imaging approach using a combined radiolabeled and fluorescent probe for guiding dissection of sentinel lymph nodes in prostate cancer [12] (Fig. 8).

### Other solid neoplasms

A variety of abstracts reported on the diagnostic utility of the main functional imaging test in oncology, FDG-PET/CT. Caobelli and coworkers from the Young AIMN Working Group [13] have nicely shown in a retrospective analysis that the predictive role of FDG-PET/CT, which was already described for the most frequent solid tumours applies also to patients with ovarian cancer. Whereas an excellent overall survival has been reported (3-year PFS > 75 %, 4-year PFS > 65 %) in patients with a history of ovarian cancer and negative PET, a markedly reduced prognosis has been described in patients with metastatic disease, as indicated by FDG-PET/CT (2-year PFS < 30 %, 4-year PFS < 10 %).

A small but increasing number of abstracts provided further evidence on the clinical utility of fully integrated PET/MRI systems. Marner and coworkers have presented initial data from an ongoing prospective clinical trial in children with malignant brain tumours [14]. PET/MRI seems to be especially relevant in children to reduce the overall radiation burden caused by medical imaging. In a sub-cohort comprising 31 patients with newly diagnosed brain tumours, PET/MRI had a major impact on the therapeutic management in two patients and a minor impact in 15 patients, respectively.

Investigations on novel targets for oncological diagnosis are essential to advance further nuclear medicine’s role in oncology, and several presentations tackled this, but only one could be highlighted as a representative example. Luo et al. [15] used a recombinant human hepatocyte growth factor (rh-HGF) radiolabelled with ^64^Cu to target c-Met, the receptor for HGF. They conjugated NOTA for radiolabelling and could show retained binding to c-Met in a set of in vitro experiments. PET imaging in tumour bearing mice revealed specific and prominent uptake of ^64^Cu-NOTA-rh-HGF in c-Met positive U87MG tumours (6.7 ± 1.8 %ID/g and significantly lower uptake in c-Met negative MDA-MB-231 tumours (1.8 ± 0.6 %ID/g). This excellent and specific c-Met targeting makes this a promising compound for assessing c-Met expression, which can be extremely useful for monitoring of responses to c-Met-targeted therapies, but can also provide a basis for targeted radionuclide therapy, which was a major outstanding scientific topic at this meeting.

In summary, significant improvements of molecular imaging for managing patients with prostate cancer and neuroendocrine tumours were shown. PSMA-PET/CT appears to be a new gold standard for staging and restaging of prostate cancer. Novel tumour specific compounds may be used for target identification and understanding the heterogeneity of solid cancers. In addition, nuclear imaging techniques appear to enter the operating room soon.

## Oncology: therapy

### Preclinical highlights

A large number of preclinical presentations included work on new targets, radiopharmaceutical tools to improve targeting efficiency, new radionuclides including alpha emitters, but also investigations into mechanisms of targeting and efficacy of radionuclide therapy approaches, three examples are representative for the high research activity in this field.

**Fig. 9:**
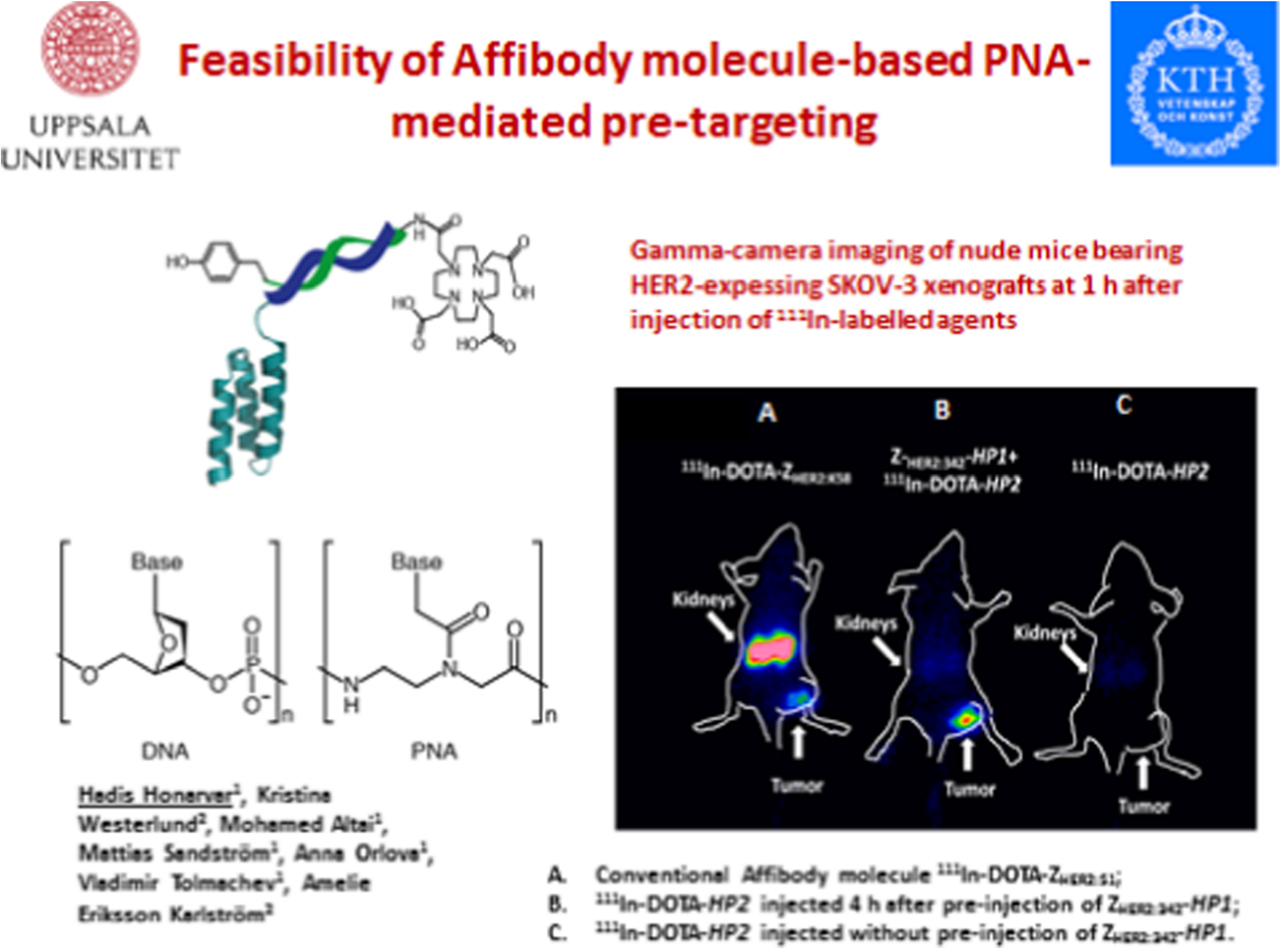
Depiction of the presentation of Honarvar [16]. The imaging has been done from SKOV-3 xenograft bearing mice using clinical gamma camera. All animals were injected with 650 kBq of a radiolabeled compound 1 h before the image acquisition. A single image of all animals was simultaneously acquired. Left mouse: animals were pre-injected with 100 μg ZHER2:342-SR-HP1 4 h prior to injection of 1 μg ^111^In-HP2. Middle mouse: ^111^In-HP2 was injected with ^111^In-HP2 without pre-injection of ZHER2:342-SR-HP1. Right mouse: animals were injected with 2 μg ^111^In-DOTA-ZHER2:K58 (650 kBq)

This year’s Marie Curie award paper from Honarvar et al. [16] dealt with the affibody molecule-based PNA-mediated pre-targeting (Fig. 9). Radiolabelled affibodies have shown great promise to serve as a platform for antigen targeting: however, they have high kidney uptake and retention, limiting their potential for radionuclide therapy. Honarvar et al. used in vivo hybridisation based on peptide nucleic acids (PNA)-mediated pretargeting to modify and thereby improve pharmacokinetics. Preinjection of PNA conjugated anti-HER2 affibody followed by ^111^In-labelled complementary DOTA-PNA resulted in higher and highly specific tumour uptake, combined with dramatically reduced kidney retention in a mouse tumour model, as compared to the ^111^In-labelled affibody itself. This novel pretargeting approach enables specific delivery of radionuclides to tumours providing improved radiometal concentration in tumours and reducing non-target accumulation applicable to affibodies, but with the potential to be more widely applied for targeting vectors for therapeutic applications.

Pouget et al. [17] investigated the relative contribution of direct and indirect (bystander) effects in the therapeutic efficacy of alpha emitting ^212^Pb-labeled mAbs in small volume peritoneal carcinomatosis model. In a complex series of in vivo and in vitro experiments comparing anti-CEA and anti Her2 MAbs they showed that despite the strong direct effects of ^212^Pb-labeled mAbs in killing tumour cells, there is a strong contribution of the bystander effects resulting in good therapeutic results even when mAbs show a high heterogenous tumour distribution. More studies like these are warranted to increase our understanding in therapeutic efficacy of specifically targeted radiopharmaceuticals for therapy to optimize the tracer design, select the most effective and safe radionuclides and direct the timing and activity of administration.

The optimal selection of targets for radionuclide therapy approaches also is dependent on tumour grade and type. Dalm et al. [18] investigated the clinical relevance of targeting SSTR2, GRPR, and CXCR4 with radioligands for imaging and therapy in breast cancer samples. They established a correlation of mRNA levels (PCR) and receptor expression of SSTR2 and GRPR (autoradiography) and subsequently investigated mRNA levels of SSTR2, GRPR, and CXCR4 in 915 clinical breast cancer samples, which they associated with known clinicopathological factors and prognosis. They found a positive correlation between high GRPR/SSTR mRNA levels and estrogen and progesteron receptor positive tumours indicating that GRPR and SSTR2 mediated imaging and therapy can be especially beneficial for this patient group, whereas high CXCR4 mRNA levels were correlated with receptor negative tumour indicating that triple negative breast cancers, for which effective therapy options are scarce, are an interesting subtype for application of CXCR4 radiotracers. In 1st line tamoxifen treatment a significant correlation between high GRPR mRNA levels and prolonged PFS after start of 1st line Tamoxifen treatment was established, indicating that GRPR expression has predictive value for the efficacy of tamoxifen therapy. This is an excellent example of a study that improves our understanding on how especially novel targeted radionuclide therapy approaches can be optimally translated into the clinical setting.

### Prostate cancer

**Fig. 10:**
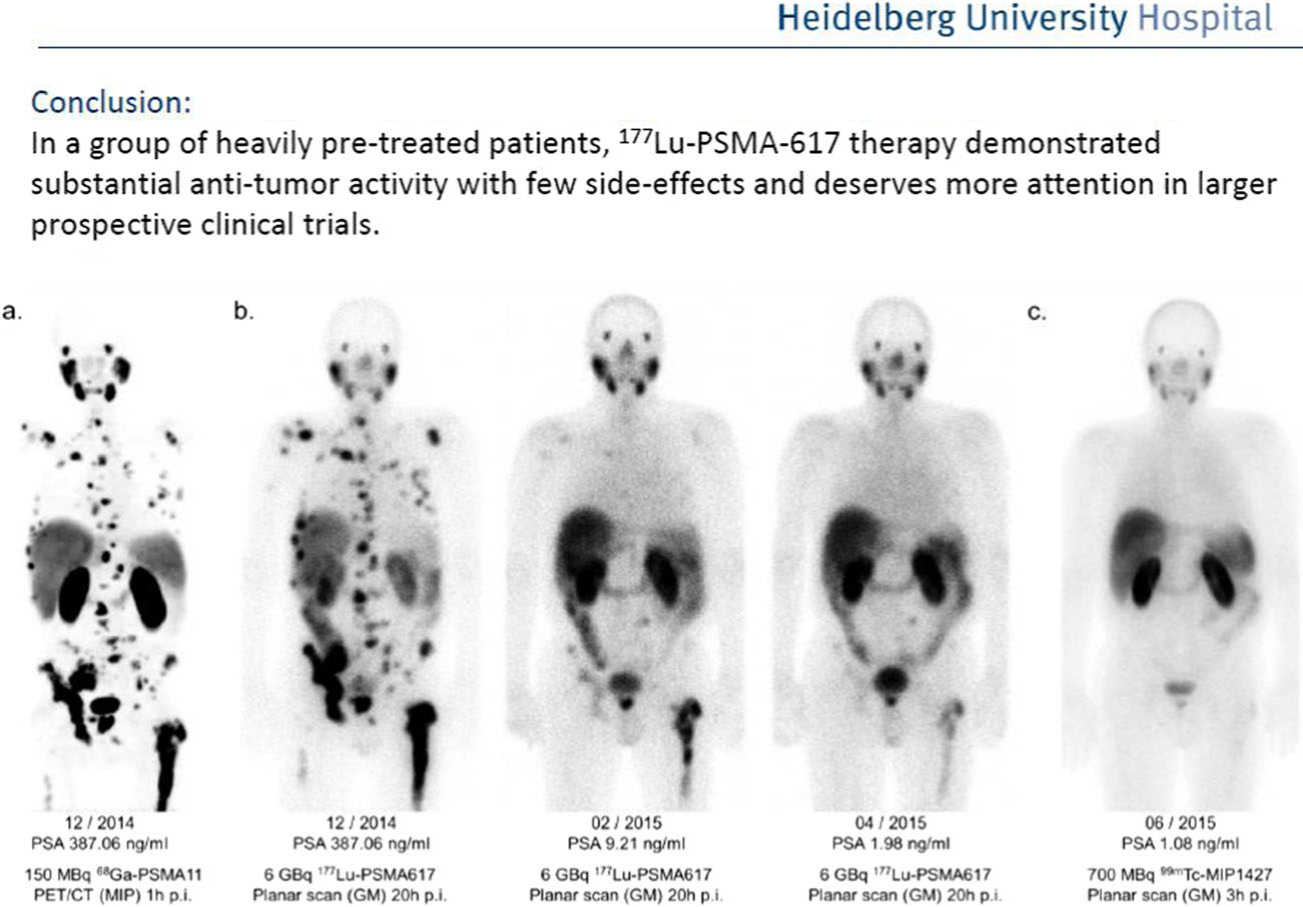
Striking therapeutic activity of ^177^Lu-PSMA617 has been presented by Kratochwil and co-workers from the University of Heidelberg [19]. (a) indicates multifocal metastatic disease to the bone at ^68^Ga-PSMA-PET/CT of a patient with progressive prostate cancer. After three cycles of treatment (b), PSMA-positive lesions completely resolved (c) and PSA-values decreased from an initial value of 387 ng/mL to 1.08 ng/mL

Striking results have been presented by Kratochwil and coworkers from the University of Heidelberg demonstrating antitumour activity of ^177^Lu-PSMA617 in metastatic hormone-refractory prostate cancer [19]. A total of 30 patients have undergone three treatment cycles in intervals of 2 months each (Fig. 10). In this heavily pretreated patient cohort, a significant drop of PSA values were reported after a single treatment cycle in 70 % of patients. After a third treatment cycle, reduction in PSA levels was >50 % in more than 70 % of patients, indicating highly effective tumour cell kill. Hence, PSMA directed radionuclide therapy represents a novel treatment strategy especially for patients with heavily pretreated metastatic disease.

Kulkarni and coworkers from the Bad Berka group have shown similar results in a related set of patients with recurrent prostate cancer [20]. After two courses of PSMA-directed treatment, a significant drop of PSA-values > 58 % has been demonstrated. These excellent and promising results for patients with advanced metastatic disease were complemented by theoretical considerations on further optimizing peptide radioreceptor therapy in prostate cancer. As an example, Kletting and coworkers [21] developed a physiologically based pharmacokinetic (PBPK) model on the most suitable ratio of radioactivity and the amount of peptide used for radiolabeling. They calculated a more rapid saturation of non-target organs (i.e., kidney, salivary glands) than in tumours, which can be the basis of further optimizing targeted tumour therapy with PSMA-ligands.

### Other solid tumours

Specific melanoma cell targeting using the benzamide derivative ^131^I-BA100 was shown by Spohn and coworkers [22]. In a preliminary study comprising 10 patients with metastatic melanoma, this novel therapeutic approach was applied on a compassionate use base. The study shows significant antitumour activity at least in a fraction of patients, supporting this interesting approach for radionuclide-based therapies in melanoma.

Selective internal radiotherapy is currently used for non-resectable primary liver tumours and liver dominant metastatic disease from solid tumours [23]. Prince and coworkers showed a new ^166^Ho-microsphere based embolization procedure, resulting in a significant delay of tumour progression in almost 60 % of patients. If this holmium based approach improves the therapeutic success already demonstrated for ^90^Y-labelled microspheres remains to be determined.

Novel targeted therapeutic approaches have been presented also for malignant brain tumours (Królicki et al.) using ^213^B-labeled DOTA-substance P [24]. The authors demonstrated that this approach is safe and well tolerated, resulting in PFS of 3.7 months. Targeting metastatic renal cell cancer using the carbonic anhydrase nine specific antibody ^177^Lu-gerentuximab was shown by Muselaers et al. from Nijmwegen [25]. Apart from transient myelotoxicity, the therapy was well tolerated and resulted in disease stabilization in 64 % of patients with progressive disease.

Whereas specific tumour targeting, safety, and tolerability has been shown in a number of novel targeted nuclear therapy techniques, overall outcome regarding disease stabilization and survival has to be assessed in prospective clinical trials for these compounds. First results from such a prospective trial were reported by Pryma et al. [26] from the United States. A phase II study evaluating Ultratrace® (iobenguane) for treatment of metastatic paraganglioma or pheochromocytoma showed its safety and tolerability. Moreover, the importance of patient-specific treatment planning by dosimetry was highlighted. Several papers have indicated that radionuclide based treatment in combination with other tumour directed therapies may improve the overall outcome. In an interesting study presented by Boni and coworkers, ^131^I-MIBG was combined with chemotherapy for treatment of high-risk neuroblastoma [27]. Whereas only preliminary data were shown, this approach seems suitable to enhance further success rates of radionuclide based treatments.

### Hematological neoplasms

**Fig. 11:**
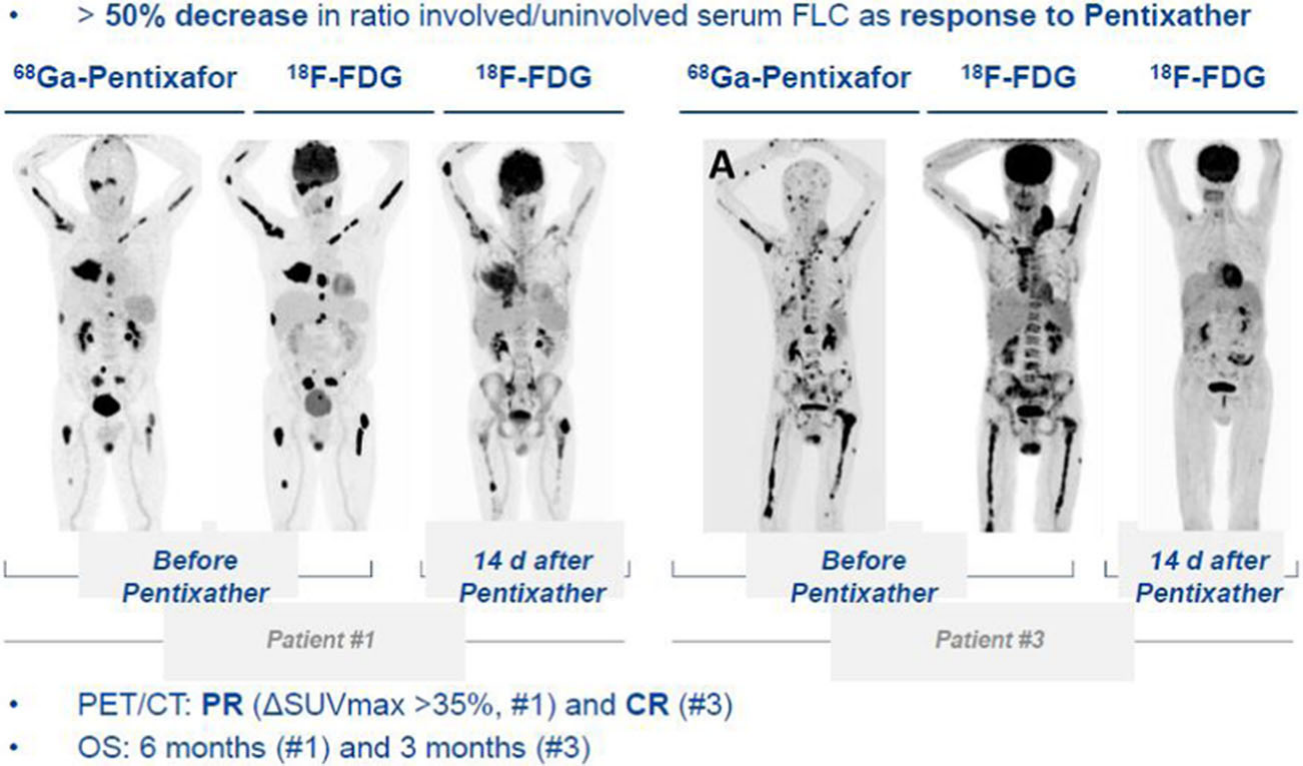
Impressive therapeutic activity of ^177^Lu-CXCR4 (Pentixather®) presented by Herrmann et al. [28]. On the left hand side, partial response of a patient with multiple myeloma lesions in the bone can be seen at FDG-PET/CT 14 days after therapy. The images on the right show almost complete remission 14d after CXCR4-directed therapy

Some striking data were reported by Herrmann and coworkers [28]. The chemokine receptor-4 (CXCR4) emerges as a novel target for imaging and treatment, particularly of hematological neoplasms. In a preliminary study comprising patients with multiple myeloma, highly effective antitumour activity was demonstrated using ^177^Lu- or ^90^Y-labelled CXCR4-specific ligands (Pentixather®), eradicating not only bone marrow related myeloma lesions, but also soft tissue and other extraosseous lesions (Fig. 11). Currently, this approach has been reported to be used also for treatment of other, CXCR4-positive solid tumours.

Whereas radioimmunotherapy targeting the CD20-antigen has been available for more than 13 years, a novel target has been used by Dahle et al. [29]. In a preclinical animal model, excellent tumour retention in lymphoma xenotransplants was demonstrated with advantages of chimeric vs. murine versions of the HH1 antibody targeting the CD37 antigen. In a related presentation by Stokke and coworkers [30], a phase1 clinical data using ^177^Lu-DOTA-antiCD37 (Betalutin®) indicated significant antitumour activity. This approach may widen the therapeutic spectrum especially in patients with CD20-refractory lymphoma.

In summary, novel theranostic concepts addressing also highly frequent tumour entities including prostate cancer, melanoma, and lymphoma were presented. Obviously, due to effective tumour cell kill and only mild to moderate side effects, an increasing role of targeted radionuclide therapies can be envisioned. Furthermore, already established radionuclide-based therapies may be further improved by using novel compounds such as SSTR-directed antagonists, further enhancing sensitivity of PET imaging and enhancing antitumour activity by increasing the delivered dose to the tumour.

## Neuroscience

Accessibility to new PET tracers especially in neurosciences depends on developments in radiochemistry. Among several submissions two presentations should be highlighted. Taddei et al. [31] presented a novel, quick, and reliable method to convert [^11^C]CO_2_ to [^11^C]CO, mediated by a [^11^C]silane derivative generated in situ and triggered by an activator. Using readily available labware, an automated system was developed giving simplified access that could lead to increased availability of [^11^C]CO chemistry to the PET community. Related to F-18, Zischler et al. [32] reported on the efficient production of PET tracers via copper-mediated radiofluorination under so called minimalist conditions. Using copper mediated aromatic nucleophilic radiofluorination of (mesityl)(aryl)iodonium salt precursors [^18^F]fluorodopamine, [^18^F]4-fluorophenylalanine and [^18^F]-DAA1106, a promising tracer for visualization of neuroinflammation, were prepared in 46–66 % radiochemical yields. Specific activities of >60 GBq/μmol were achieved. This radiofluorination method that combines the advantages of “minimalist” approach to radiolabelling with the exceptional capabilities of copper mediated aromatic nucleophilic radiofluorination, opens new ways for the efficient preparation of a number of interesting radiopharmaceuticals especially for neurolological applications.

Additionally, this meeting was characterized by a number of submissions on the development of improved ligands and preclinical characterisation of developed ligands in novel preclinical models for various highly interesting targets in neurology and psychiatry. Rami-Mark et al. [33] presented the preclinical evaluation of a new ^11^C-tracer for the norepinephrine transporter (NET), [^11^C]Me@HAPTHI. This tracer showed an excellent affinity (K_D_ = 0.21 ± 0.07nM) and selectivity (DAT/NET > 1940; SERT/NET = 9700) for the NET and high metabolic stability. In autoradiographic experiments on human brain tissue, highest uptake was observed in NET-rich regions and a concentration dependent binding displacement observed. This promising compound awaits further in vivo evaluation for translation into the clinic.

Gargiulo et al. [34] used [^18^F]DPA-714 to image translocator protein-expression (TSPO, a marker for neuroinflammation), in a mouse model of amyotrophic lateral sclerosis (ALS) via μPET/CT. In the SOD1G93A symptomatic mice, the region/frontal cortex ratio was significantly increased (*p* = 0.012) in the brainstem (2.340 ± 0.784) as compared to non-carriers wild type mice (1.576 ± 0.287). These preliminary results suggest that increased [^18^F]DPA-714 uptake can be measured with high resolution PET/CT in a mouse model of ALS in the brainstem, a region known to be the site of degeneration and increased microglial activation. Correlating with positive immunostaining of TSPO in the brain stem suggests that increased microglial activation might be the cellular counterpart of in vivo increased [^18^F]DPA-714 uptake and provides a basis for ALS imaging by means of PET.

Another TSPO targeting ligand ([^11^C]PBR28) was used by Toth et al. [35] for longitudinal imaging of acute neuroinflammation in a rat ischemic stroke model by micro-PET. They monitored the inflammatory response after transient cerebral ischemia in rats, using a recently developed rat stroke model with isolated focal cortical infarcts. [^11^C]PBR28 showed high uptake in the infarct region from day 4 with gradual decrease at later time points. The longitudinal follow-up of inflammatory response with the TSPO radioligand [^11^C]PBR28 showed significantly up-regulated TSPO binding in the infarct region from day 4, with both %SUV and binding potential opening new possibilities for studies on neuroinflammation in ischemic stroke.

[^18^F]-THK-5117 is a promising PET tracer for imaging abnormal accumulation of tau aggregates in brain as one of the hallmarks of Alzheimer’s disease. Brendel et al. [36] evaluated the radioligand [^18^F]-THK-5117 in different mouse models (P301S1, biGT2, Tau223) of tau pathology using μPET in combination with autoradiography and histopathology. They showed that Tau imaging in mice is feasible and correlates with ex vivo findings. The Tau 22 model was best suited to image pathology-related effects. This in vivo assessment of the given inter-animal heterogeneity can, therefore, potentially improve subsequent treatment studies in the future.

**Fig. 12:**
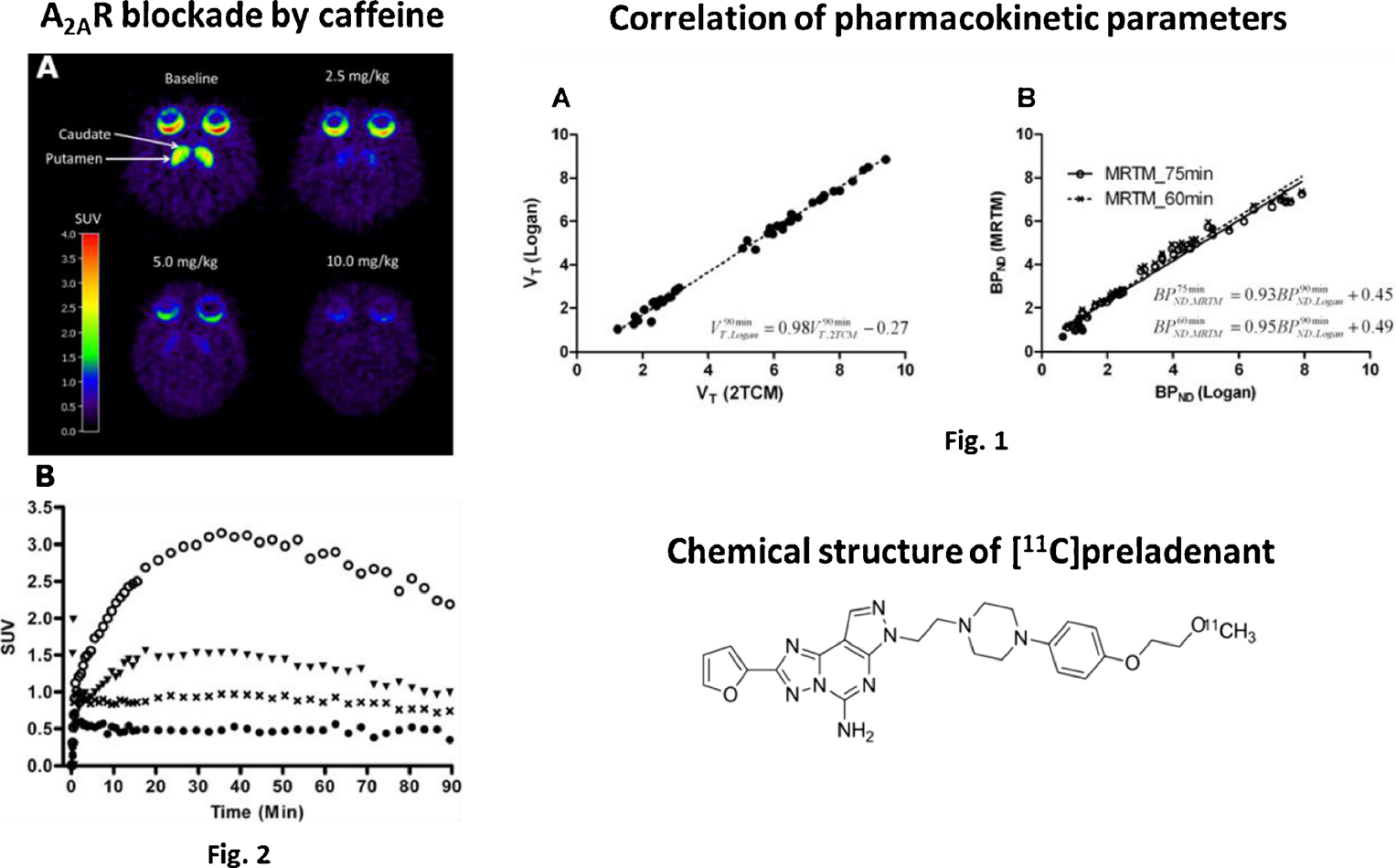
In vivo evaluation of [^11^C]preladenant for imaging of adenosine A2A receptors in the conscious monkey [37]. The tracer kinetics in the monkey brain is well described with the 2-tissue compartment model and Logan graphic analysis with metabolite corrected plasma input function. Figure 1a shows the good correlation in distribution volume between 2-tissue compartment model and Logan plot. Binding potential was estimated with multilinear reference tissue model. The value was in agreement with binding potential calculated from distribution volume ratio (DVR)-1, as is shown in Fig. 1b. Caffeine pretreatment reduced the tracer uptake in striatum in a dose-dependent manner (Fig. 2). Figure 2b shows that the standardized uptake values in striatum (putamen and caudate) correspond to baseline (open circle), and 2.5 (triangle), 5 (cross), 10 (closed circle) mg/kg caffeine pretreatment (A2AR occupancy 52, 63, and 81 % for 2.5, 5, and 10 mg/kg caffeine, respectively)

The in vivo evaluation of a novel ligand ([^11^C]preladenant) for the adenosine A_2A_ receptor, a therapeutic target in several neurologic and psychiatric disorders, was presented by Zhou et al. [37]. They imaged [^11^C]preladenant in conscious monkeys; caffeine pretreatment reduced the tracer uptake in striatum in a dose-dependent manner. Kinetic modelling allowed to estimate binding potential in striatum and to quantify A2AR density (Fig. 12). [^11^C]Preladenant PET was suitable to noninvasively quantify A2ARs in monkey brain and holds great potential to translate this into the clinical setting.

**Fig. 13:**
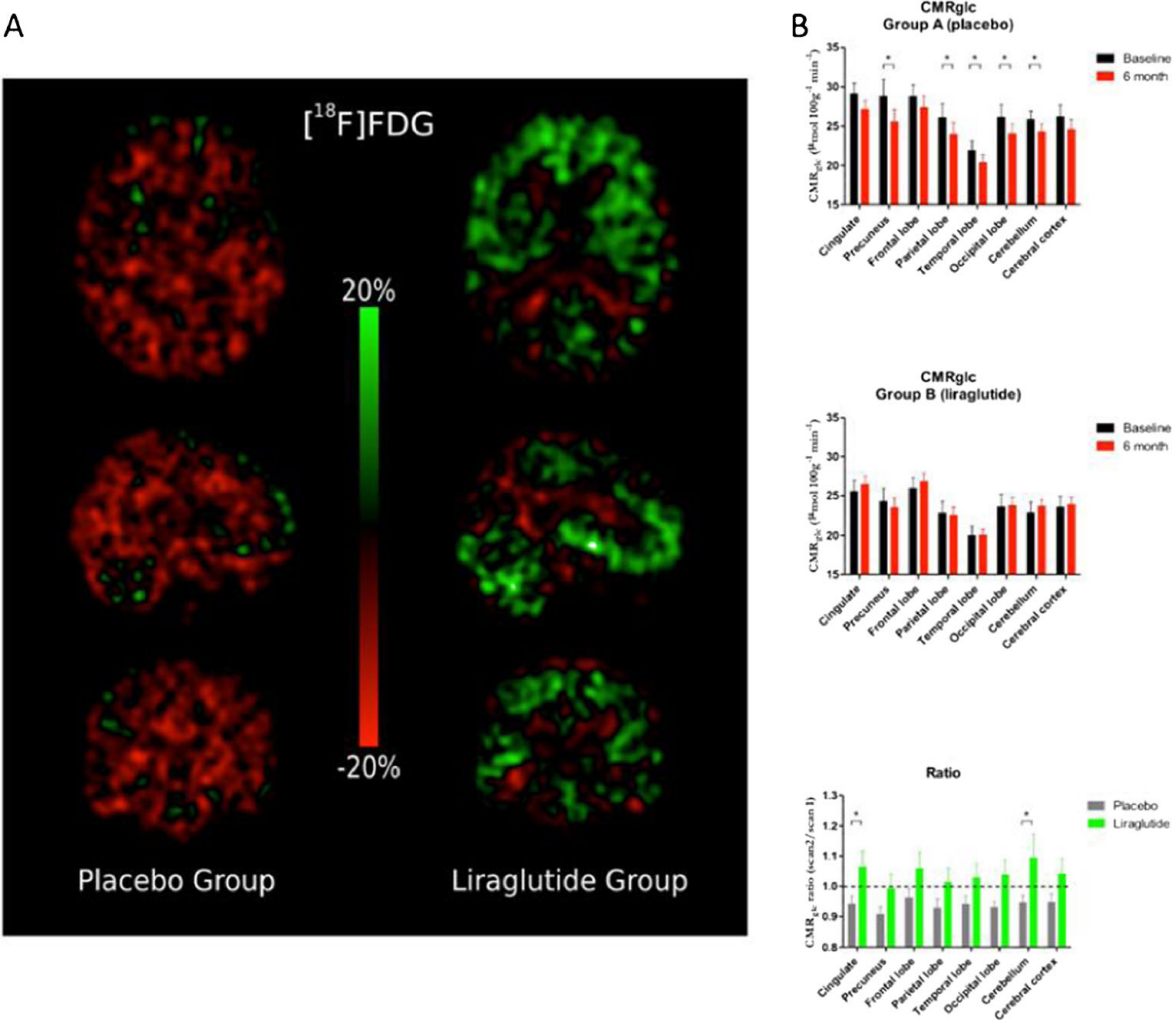
In AD patients, liraglutide treatment prevented the decline of CMRglc seen in placebo-treated patients where it is considered to signify cognitive impairment, synaptic dysfunction, and disease progression [38]

Numerous excellent papers were also found in the human application in neurosciences. Gejl et al. [38] presented results from a randomized, placebo-controlled, double-blinded clinical trial investigating the effect of the GLP-1 analogue liraglutide on the decline of brain glucose metabolism in Alzheimer’s disease. In this PET study they compared the results of ^11^C-PIB (as marker for amyloid plaque) and FDG (as a surrogate marker of synaptic dysfunction and neuronal activity) imaging in a group treated with liraglutide (*n* = 18) or with placebo (*n* = 20) in a 26-week interval. In both groups, amyloid plaque deposition significantly increased, but no difference between the groups was found. Cerebral metabolic rate of glucose (CMRglc) measured by FDG-PET, however, declined significantly in the placebo group, whereas liraglutide medication resulted in a numerical, albeit insignificant, increase of CMRglc after the 6 months of treatment (Fig. 13). Liraglutide treatment, therefore, prevented the decline of CMRglc seen in untreated Alzheimer’s patients where it signifies cognitive impairment, synaptic dysfunction, and disease evolution.

New PET tracers PET could play an increasing role also in Parkinson’s disease, allowing in particular improved means of quantification. Sonni et al. [39] reported on the applicability of a simplified quantification of the dopamine transporter using the novel dopamine transporter ligand [^18^F]FE-PE2I. In 10 PD patients and 10 control subjects they identified the optimal acquisition time window between 16.5 and 42 min to the determine specific binding ratio (SBR) that is in closest agreement with the binding potential. Despite an underestimation of BPND, and possibly decreased sensitivity to detect nigrostriatal deficit, the SBR can be viewed as a valid quantification method for DAT using [^18^F]FE-PE2I for diagnostic purposes, since it provides similar differentiation as BPND between normal and Parkinson’s patients, thereby allowing translation of quantitative PET dopamine transporter imaging into a routine clinical setting.

The clinical impact of amyloid PET imaging has been a matter of debate over the last years. In a highly interesting clinical study Pontecorvo et al. [40] investigated the impact of florbetapir (^18^F) PET amyloid imaging on patient management and outcome. This randomized, controlled, multicenter, international trial included 618 patients with MCI or dementia, where AD was considered a possible cause (<85 % certain). Patients underwent a florbetapir PET scan and were then randomized to either immediate (*n* = 308) or delayed (1 year, *n* = 310) feedback regarding amyloid status (positive: Aβ+, negative: Aβ-). After 1 year, treatment was changed in 32.3 % in the group with immediate feedback, whereas only in 8.2 % in the delayed feedback group. Ach-inhibitor medication was 74 % (Aβ+) vs. 23 % (Aβ-) in the immediate as compared to 56 % (Aβ+) vs. 41 % (Aβ-) in the delayed feedback group. This is the first large multicenter randomized controlled study of the clinical impact of amyloid PET and may help guide and strengthen the use of amyloid PET imaging.

Overall, this meeting confirmed the high translational potential of nuclear medicine in neurosciences. Novel tools in radiochemistry provide the basis for better accessibility of radiotracers, characterisation in novel disease models open new opportunities in preclinical research, also strengthened by improved tracers for known targets. A number of targets of high interest in neurology and psychiatry are now accessible for PET imaging including A2A, TSPO, NMDA, and many more giving great new opportunities for clinical application and research on nuclear medicine and particular PET in neurosciences.

## Cardiovascular

**Fig. 14:**
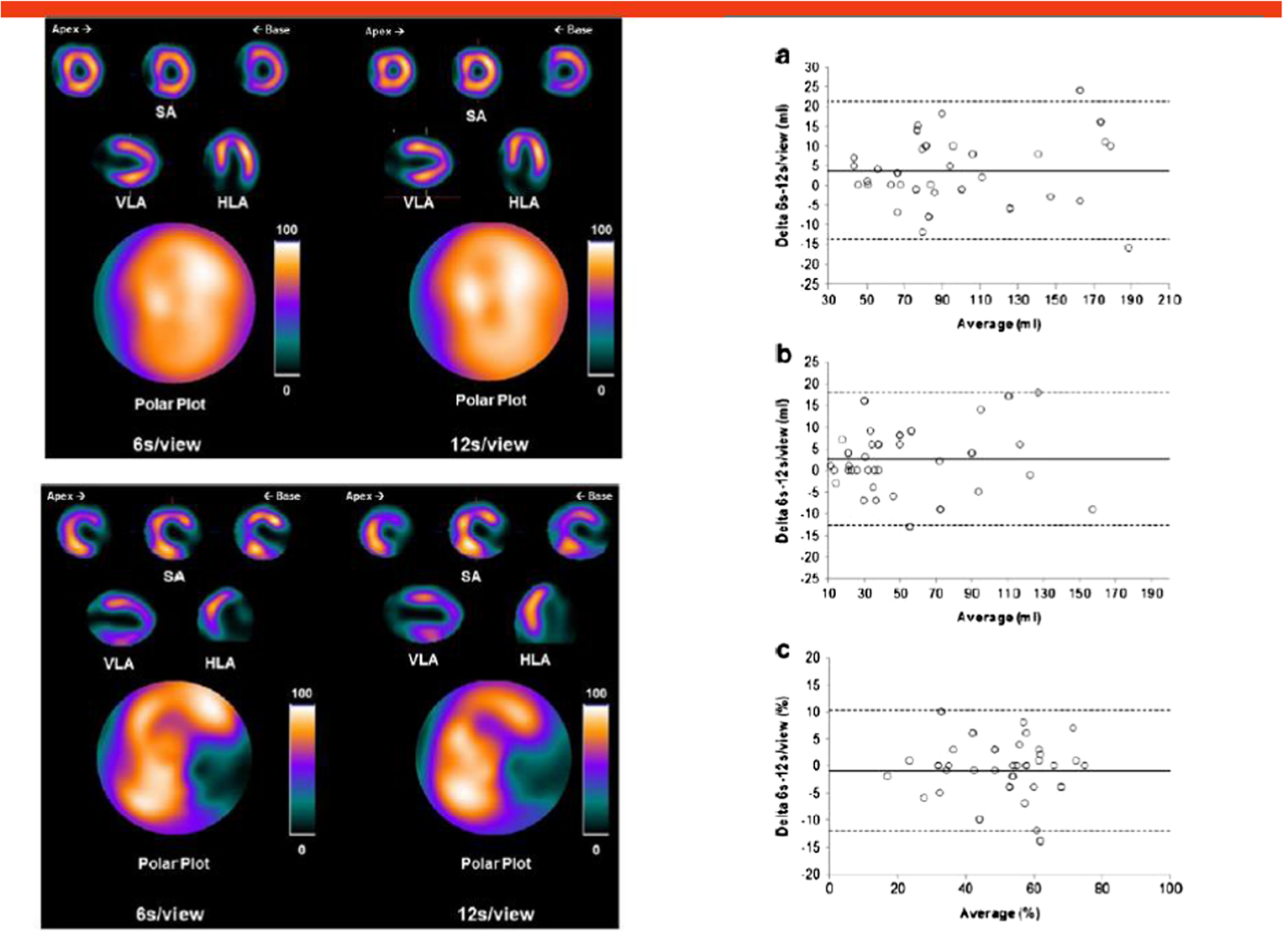
Caobelli and coworkers [41] assessed the feasibility of reducing scanning time for myocardial perfusion scanning by one quarter and one eighth using the IQ SPECT system. They have shown somewhat reduced image quality (left images, upper and lower panel) at 6 s/view as compared to 12 s/view acquisition time (right images, upper and lower panel), whereas summed scores were equivalent using both acquisition protocols

In the cardiovascular tract, a significant fraction of submitted abstracts focused on further improvement of myocardial perfusion imaging. An interesting contribution by Caobelli and coworkers [41] has demonstrated significant reduction of the scan duration using IQ-SPECT. Myocardial perfusion imaging was performed using reduced acquisition time of just one eighth or one quarter of the standard scanning time (Fig. 14). The authors adequately showed that image quality was somewhat reduced, but did not affect diagnostic accuracy.

Despite savings in acquisition time, reduction of radiation burden and enhanced motion correction were also major issues, which have been addressed by several contributions. In a relevant study presented by Liu and coworkers from Sydney, submillisievert stress perfusion imaging was evaluated [42]. Using a novel cadmium zinc telluride SPECT camera in combination with a CT device (CZT SPECT/CT) and reduced activity of only 100 MBq, a superior image quality was achieved. Data derived from the calcium scoring CT were used for correction of attenuation artefacts.

Motion correction using a similar device was presented by Redgate and coworkers [43]. Adequate motion correction could be performed by summing reconstructed 33 images. They showed that this is technically feasible and accurate, improving outcome of cardiac studies.

An interesting study was provided by Rijnierse et al. from Amsterdam [44]. Using multimodal imaging techniques, the role of myocardial perfusion imaging, detection of sympathetic denervation and scar size were used to assess their predictive role on detecting inducible ventricular arrhythmia in patients with ischemic cardiomyopathy. [^15^O]H_2_O-PET for myocardial perfusion imaging and [^11^C]HED-PET for innervation imaging has been performed. Of interest, impaired hyperemic perfusion of the left myocardium was the only independent predictive parameter of ventricular arrhythmia. Scar size, sympathetic denervation, and an obvious innervation-perfusion mismatch were of less significance. Another relevant study related to coronary artery disease was presented in the technology track. Kan and coworkers assessed the frequency of side branch occlusion after stenting of the ramus descendens anterior (RDA) [45]. Using [^13^N]NH_3_-PET for myocardial perfusion scanning, they showed that side branch occlusion occurred in a relevant proportion of the total patient cohort (38 %). However, defects were usually small and diminished completely at rest perfusion scanning. Knowledge of side branch occlusion in a relevant fraction of patients undergoing RDA stenting is relevant for interpreting myocardial perfusion scans in daily clinical practice.

Despite coronary artery disease, other cardiac illnesses have been in the focus of a variety of contributions. In a preliminary study, Sgart and coworkers have shown that FDG-PET/CT in combination with cardiac MRI could be used not only to identify cardiac involvement of sarcoidosis, but also to monitor response to treatment [46]. Dellavedova and coworkers from Milan have shown that the uptake of FDG in the vessel wall of patients with large vessel vasculitis can be used as a prognostic marker, identifying patients with a more complicated course of the disease or disease progression [47]. This information could be used by clinicians to further guide therapeutic interventions.

**Fig. 15:**
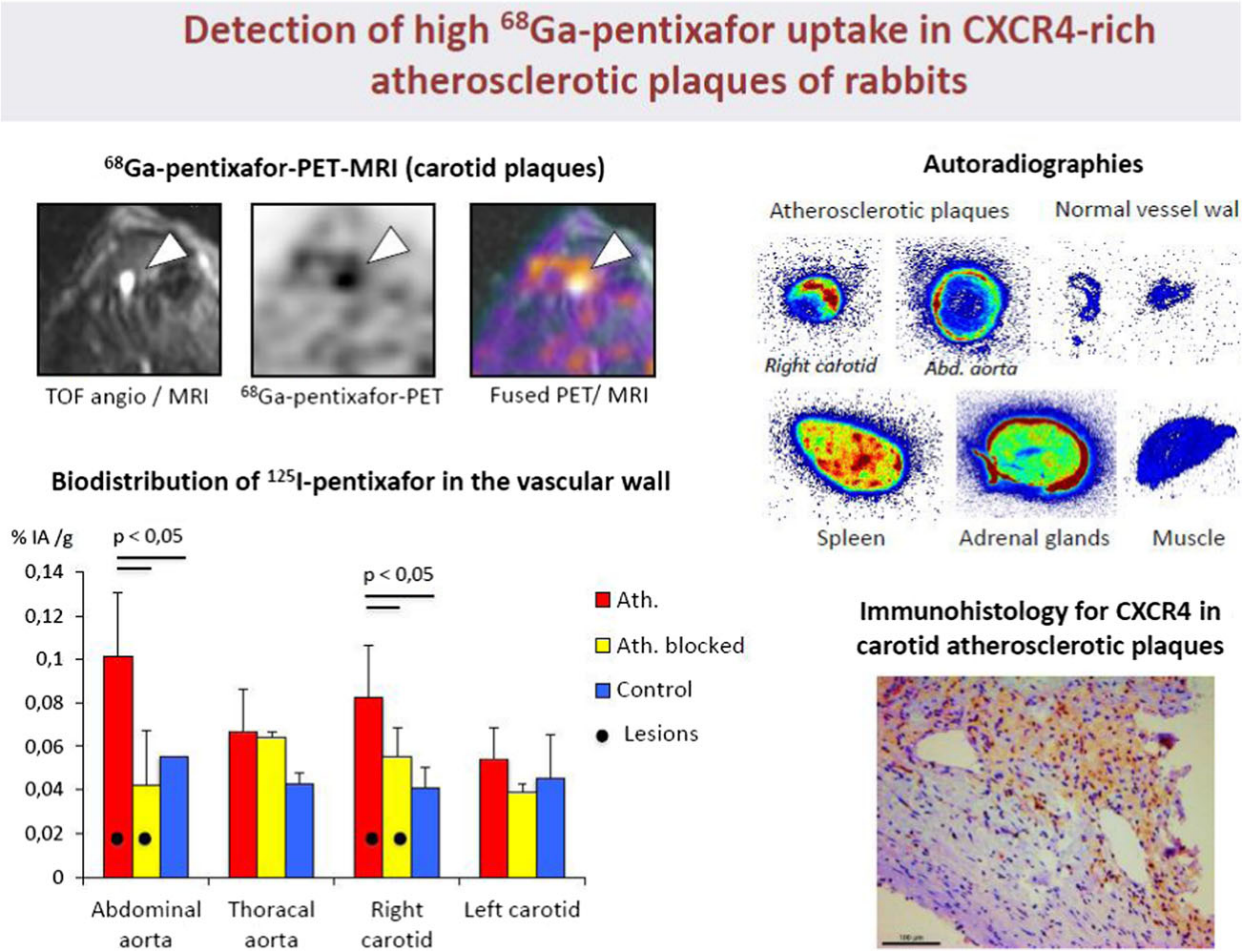
Hyafil et al. [48] presented innovative work using the novel chemokine receptor 4-specific radiotracer Pentixafor® for imaging vulnerable plaques. In a canine model of vascular inflammation, CXCR4-specific retention of the radiotracer was shown in atheroscleoritic plaques, whereas no tracer uptake was detected in the normal vessel wall. Upper left: intense uptake of pentixafor in the right atherosclerotic carotid artery (PET-MRI). Biodistribution studies confirmed specific binding of pentixafor in atherosclerotic plaques, which was inhibited by blocking after co-injection of a CXCR4 inhibitor (lower left panel). Higher pentixafor uptake (red color) was detected in atherosclerotic plaques as compared to control arteries (upper right panel). Intense expression of CXCR4 in atherosclerotic plaques (brown staining) was identified in carotid plaques bi immuno-histology in the rabbit model (lower right panel)

Imaging of vulnerable plaque represents a so far unmet clinical need. The CXCR4-directed PET probe ^68^Ga-Pentixafor® developed by H.J. Wester at TU Munich has been assessed in a rabbit model of arteriosclerosis by Hyafil and coworkers [48]. The authors have shown specific retention of the radiotracer in experimental arteriosclerotic plaques of the carotid artery. CXCR4-specific retention was proven also by an adequate control (non-inflamed vessel wall) and correlation to corresponding immunohistochemical sections stained for CXCR4-expression (Fig. 15).

In summary, a variety of presentations focused on further optimization of cardiac imaging by reducing the radiation exposure to the patient, reducing scanning time and further optimizing motion correction. Of interest, several contributions addressed molecular imaging of inflammation in the cardiovascular system. Specifically, large vessel vasculitis and cardiac sarcoidosis were major diseases of interest which were addressed also by novel radiopharmaceuticals targeting chemokine receptor expression.

## Conventional nuclear medicine: miscellaneous

Besides the major topics oncology, cardiology, and neurosciences, also almost 200 abstracts accounted for topics outside this mainstream and covered a great variety of diseases. An example of research on a novel thyroid targeting agent was presented by Galli et al. [49], who reported on further improvements towards radiolabelled TSH analogues. They compared two new ^99m^Tc-labelled superagonist rhTSH analogues for imaging poorly differentiated thyroid cancers. ^99m^Tc-TR1402 proved superiority over ^99m^Tc-TR1401 and ^99m^Tc-thyrogen with high affinity to the receptor, a high focal uptake in mice bearing TSHR-cells and excellent imaging properties in dogs with spontaneous intra-glandular papillary thyroid cancer, in which TSHR expression was confirmed by immunohistochemistry, opening new perspectives for pre-surgical TSHR-based staging of thyroid cancer, for diagnosis of radioiodine negative cancer remnants, local relapse, and/or distant metastases.

**Fig. 16:**
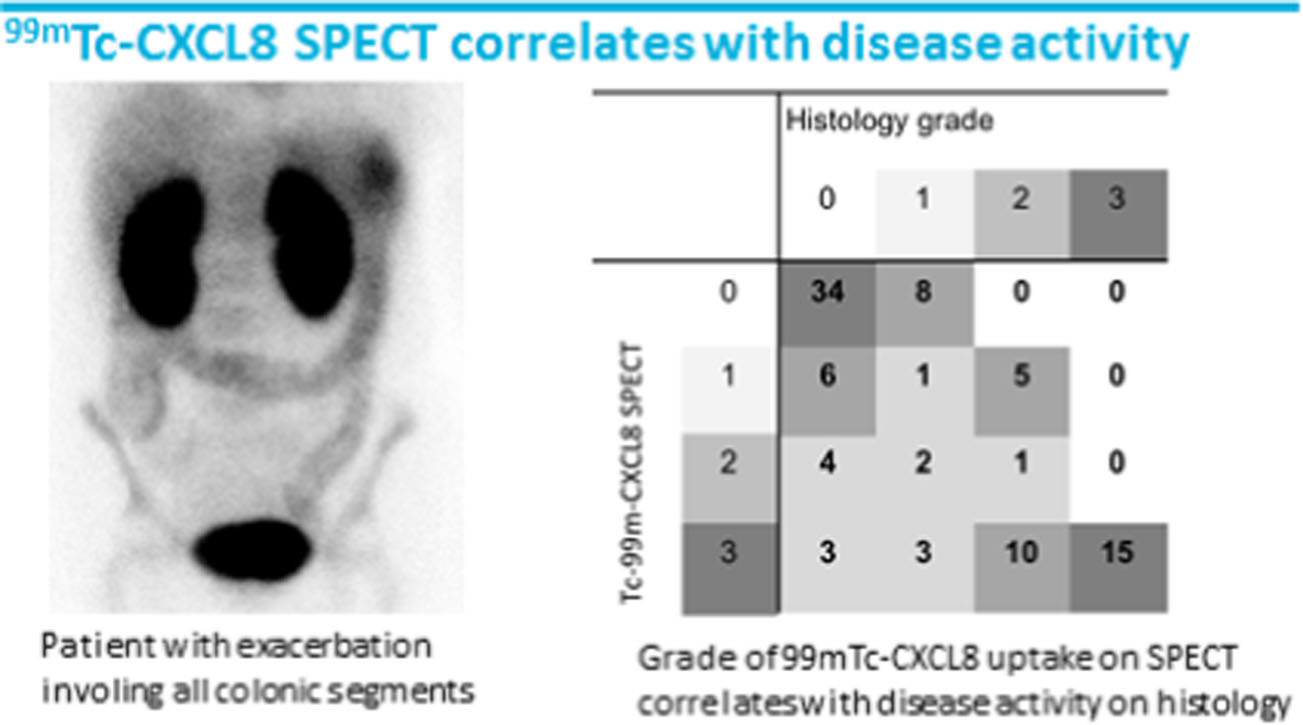
^99m^ Tc-CXCL8 in inflammatory bowel disease [50]. Left: a patient with ulcerative colitis in an exacerbation phase of the disease, showing severe disease activity involving all segments of the colon, from ascending colon (but not terminal ileum), transverse colon through the sigmoid. Right: a per-segment analysis of the grading according to the ^99m^ Tc-CXCL8 SPECT, measured as compared to bone marrow, which has a stable level of ^99m^ Tc-CXCL8 uptake during both exacerbations and quiet phases of disease, and according to the pathologist. In grey scale, the number of evaluated segments are shown. At both ends of the spectrum, no disease activity or severe, the correlation was excellent, no false negatives, a few false positives mainly located in the ascending colon

An excellent clinical study using ^99m^Tc-CXCL8 SPECT to image disease activity in inflammatory bowel disease was reported by Aarntzen et al. [50]. In a total of 30 patients (17 Crohn’s disease, 14 ulcerative colitis) uptake of ^99m^Tc-CXCL8 in intestinal lesions was significantly increased during exacerbations, as compared to scans performed during follow-up. They demonstrated that ^99m^Tc-CXCL8 SPECT has an excellent diagnostic accuracy on a per-segment analysis, as compared to endoscopy and is a non-invasive whole body alternative for endoscopy to monitor disease activity in inflammatory bowel disease (Fig. 16).

Also, well-established radiopharmaceuticals and nuclear medicine imaging techniques still have a strong clinical utility, as shown in a number studies presented at the meeting. Medaer et al. [51] looked at the impact of colonic transit scintigraphy on clinical decision making in severely constipated children. Ten patients with chronic treatment-resistant functional constipation received an ^111^In-DTPA colonic transit scintigraphy. The results of the scintigraphy lead to a change in management in 9/10 patients and had therapeutic consequences 7/10 patients. This preliminary study demonstrates the added value of colonic transit scintigraphy, an old technique, in the investigation of functional constipation in children and the high impact on clinical decision making.

## Physics and instrumentation

Last, but certainly not least also physics and instrumentation contributed significantly to the scientific excellence of this meeting. A number of great innovations were reported regarding to new instruments opening many opportunities both for clinical and preclinical imaging.

**Fig. 17:**
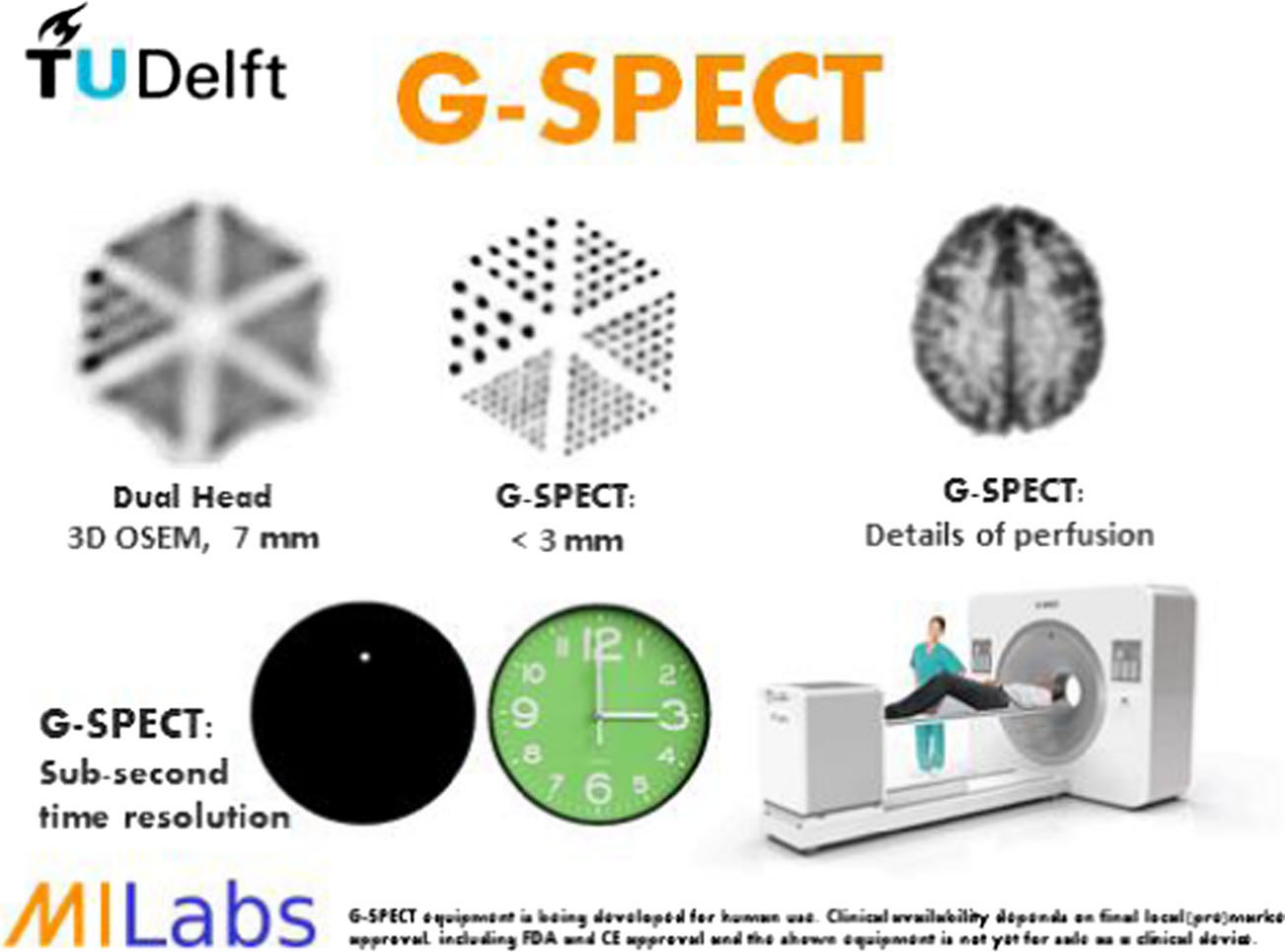
Properties of a novel SPECT system (G-SPECT) [52]. Left top: Comparison of phantom imaged on a conventional double head camera as compared to G-SPECT: equal acquisition time and dose shows superb resolution for G-SPECT. Right top: Hoffman brain phantom shows uptake in small structures. Bottom: A clock was put in the scanner with 0.1 MBq ^99m^ Tc on the second hand. This shows that a tremendous speed can be obtain with G-SPECT, opening up new possibilities for fast dynamic studies

Beekman et al. [52] developed a new full-ring stationary clinical SPECT system, G-SPECT-I, based on a stationary ring consisting of nine large field-of-view cameras with 595 × 472 mm NaI crystals, a 3D stage that allows optimal sampling during scanning, and an interchangeable nonagon-shaped collimator containing 54 pinhole apertures and with a bore diameter of 398 mm that makes the system suitable for brain, extremity, or pediatric imaging (Fig. 17). Using resolution phantoms, a comparison was made with dual head SPECT with low energy high resolution collimators and 3D OSEM. Smallest rods resolved with G-SPECT-I where 2.5 mm as compared to 7 mm with a conventional dual head SPECT. Peak sensitivity of G-SPECT-I was 630 cps/MBq compared to 182 cps/MBq for dual head; dynamic imaging with time frames of <10 s was possible for focused scans. This excellent spatial and time resolution combined with a high sensitivity potentially opens new opportunities for SPECT imaging.

Several papers reported on new technical developments for preclinical imaging, reducing size, or improving resolution or sensitivity. Segbers et al. [53] showed first images with the Mediso nanoScan preclinical SPECT/MR system. High resolution SPECT imaging (<0.9 mm using a multi-pinhole SPECT subsystem with four rotating 9.5 mm NaI detectors) and accurate quantification of tumour uptake within 11 % was possible providing fused MRI images (using a 1 Tesla magnet) with clear anatomical details in normal animals and tumour xenotransplant models.

Also, new clinical applications can be expected in the future based on new imaging technologies. The general idea and the functionality behind a so-called Real-time Handheld Emission Spot Allocator (rthESA) for Simultaneous Fusion of Nuclear Imaging with Ultrasound was presented by Kühnel et al. [54]. The rthESA prototype to allocate a radioactive spot source is based on electronic collimation, capable of assessing the distance of the source from the detector, avoiding the need for classic reconstruction thereby allowing real-time imaging. This was physically combined with an ultrasound probe and both devices depict the same plane in an imaging model. Such an imaging modality would open new ways for imaging in a variety of clinical challenges.

Also in image reconstruction a number of new approaches were reported. Engberg et al. [55] used voxel-wise analysis for multiparametric characterization of early tumour response by PET/MR in patients with lung cancer. Eight patients with histologically or cytologically confirmed lung cancer underwent simultaneous FDG-PET/MRI (Siemens mMR) prior and different days after the first or second cycle of chemotherapy. Multiparametric imaging with simultaneous FDG-PET and DW-MRI in lung cancer patients allowed identification of a responding pattern by voxel-wise GMM analysis already 24 h after chemotherapy, potentially enabling a rapid shift to a more efficient therapy in nonresponding patients.

Eberlein et al. [56] analysed DNA double strand break biomarkers γ-H2AX and 53BP1 in lymphocytes of 16 patients during their first peptide receptor radionuclide therapy and 20 patients during radioiodine therapy of differentiated thyroid cancer. The number of radiation induced foci (RIF) as a function of time was characterized by a linear dose-dependent increase and a multi-exponential decay function characterizing different rates of DNA repair. If the absorbed dose to the blood exceeded 20 mGy in the first hour, on-set of a fast repair component (only observed in DTC patients) resulting in a bi-exponential repair function was observed; otherwise a mono-exponential function better described the repair of the double strand breaks. This study revealed a threshold dose of 20 mGy within the first hour for the induction of a fast repair component after in vivo double strand break induction.

Finally, a study is highlighted dealing with radiation dose during routine clinical imaging. Johnsen et al. [57] studied the cumulative radiation dose received by diagnostic imaging in 20 children and young adults diagnosed with primarily operable Ewing Sarcoma diagnosed in Norway in the period 2005 to 2012, without metastases or residual disease in a follow up period of 1 year after diagnosis. The estimated mean cumulative radiation dose of patients received during the first year after diagnosis was 39 (6–86) mSv, the contribution from nuclear medicine imaging was 22 mSv. PET/CT imaging contributed to only 15 % of the total radiation dose the study revealed a wide range of diagnostic imaging modalities and examination performed on this patient group. This is an excellent example of studies required to prove the suitability and rationale of all new innovations we foresee with nuclear medicine techniques in the years to come to use them in the best way for our patients.

## Conclusion

The 2015 annual congress of the European Association of Nuclear Medicine is an excellent example for the increasing number of functional and molecular imaging techniques becoming available in recent years. In addition, the clinical relevance of nuclear medicine is fueled by a number of targeted, radionuclide based treatment approaches. Effectiveness regarding diagnostic accuracy as well as therapeutic activity has been shown for many clinical scenarios. Further evidence of the clinical utility has been provided by joint forces of basic scientists including physicists, chemists, biologists, pharmacists, engineers, as well as technicians and medical doctors.

Bringing all these specialties together and addressing a plethora of yet unmet clinical needs is a heroic work and many “superhero scientists” have been identified at the 2015 EANM meeting contributing to various research activities. Collaborations with superheroes also of other medical disciplines, including—among others—oncologists, cardiologists, neurologists, radiation oncologists, and surgeons, will pave the way for the widespread acceptance of these novel imaging techniques and treatments, becoming a future standard for imaging and therapy of our patients.
